# Proactive case-finding and risk-stratification in people at risk of chronic liver disease in Greater Manchester: a cost-effectiveness analysis

**DOI:** 10.1136/bmjph-2025-003480

**Published:** 2026-07-13

**Authors:** Gabriel Rogers, Stephanie Landi, Huw Purssell, Tonia Momoh, Sol Yates, Oliver Street, Karen Piper Hanley, Neil Hanley, Varinder Athwal, Katherine Payne

**Affiliations:** 1 Manchester Centre for Health Economics, The University of Manchester Faculty of Biology Medicine and Health, Manchester, UK; 2 The University of Manchester Faculty of Biology Medicine and Health, Manchester, England, UK; 3 Manchester University NHS Foundation Trust, Manchester, UK; 4 College of Medicine and Health, University of Birmingham, Birmingham, UK; 5 University Hospitals Birmingham NHS Foundation Trust, Birmingham, UK

**Keywords:** statistics and numerical data, Mass Screening, Program Evaluation

## Abstract

**Introduction:**

We urgently need innovative strategies to combat a growing epidemic of chronic liver disease (CLD). Integrated Diagnostics for Early Detection of Liver Disease (ID-LIVER) was a collaborative project aiming to improve detection of reversible-stage CLD in a region with high prevalence of critical risk factors. This study assesses the cost-effectiveness of different ways to identify people with significant CLD (defined as METAVIR stage F2 or higher, using liver stiffness of ≥8 kPa on transient elastography as a proxy measure). Strategies of interest include proactive case-finding in the community (supplementing a reactive pathway where hepatology referrals are passively received from primary care) and/or risk-stratification (using Fibrosis-4 (FIB-4) or ID-LIVER-Machine Learning (ML)—a novel machine-learning risk-stratification tool).

**Methods:**

We developed a state-transition decision-analytic model estimating lifetime healthcare costs (2023/2024 GBP) and quality-adjusted life-years (QALYs) associated with six alternative strategies for case-finding and risk-stratification. We simulated cohorts of people with alcohol-related liver disease and metabolic dysfunction-associated steatotic liver disease. We populated the model with data collected in ID-LIVER, supplemented by parameters from the literature and routine data sources. We estimated incremental cost-effectiveness and performed deterministic and probabilistic sensitivity analyses.

**Results:**

Any case-identification strategy costing ≤£3300 per person with significant CLD identified would meet English cost-effectiveness thresholds (£20 000/QALY). In our decision set, the cheapest strategy is to use FIB-4 in the reactive-only population; however, this misses 43.6% of people with significant CLD. ID-LIVER-ML (using a cut-off of 0.4) generates more population health at a reasonable cost (£10 498/QALY gained). Introducing proactive case-finding generates further health benefits, costing £12 952/QALY gained. Using ID-LIVER-ML in the proactive-and-reactive population has the highest probability of maximising cost-effectiveness, when valuing QALYs at £20 000.

**Conclusions:**

Smart methods of case-finding and risk-stratification identify people with significant CLD in the community and are likely to represent good value for money in England.

**WHAT IS ALREADY KNOWN ON THIS TOPIC**

- Steatotic liver disease is a leading and growing cause of premature mortality in England. But finding people who would benefit from a diagnosis while making efficient use of NHS resources is challenging.

**WHAT THIS STUDY ADDS**

- Judged by usual English standards, it is good value to supplement reactive hepatology referrals with proactive case-finding of people with alcohol-associated and metabolic-associated liver disease in the community. The strategy that provides greatest benefits at acceptable cost is to combine proactive case-finding with risk-stratification using a new machine-learning-based model, Integrated Diagnostics for Early Detection of Liver Disease-Machine Learning.

**HOW THIS STUDY MIGHT AFFECT RESEARCH, PRACTICE OR POLICY**

- Proactive case-finding of alcohol-associated and metabolic-associated liver disease has the anticipated benefits for the person and can be achieved in ways that represent good value for the system. The common practice of risk-stratifying referrals using FIB-4 misses meaningful numbers of people with significant liver disease.

## Introduction

It almost seems to go without saying that early detection of chronic liver disease (CLD) will improve patient outcomes and deliver better value to health systems. A substantial number of people live with undiagnosed CLD, which often remains asymptomatic until it becomes life-threatening.^1^ Identifying these cases earlier could improve health outcomes and reduce long-term healthcare costs. However, finding people who would benefit from a diagnosis of CLD while making efficient use of NHS resources is challenging. Early detection initiatives have costs and, without targeted approaches, hepatology services risk becoming overwhelmed by referrals of people who ultimately do not have clinically significant disease. While some local commissioners have established referral pathways from primary care, fewer have implemented proactive case-finding strategies for at-risk individuals in the community.^2^

We can attribute around 90% of CLD mortality in England over the last two decades to adverse lifestyle-related and socioeconomic factors, with the most common causes alcohol-related liver disease (ARLD) and metabolic-dysfunction-associated steatotic liver disease (MASLD).^3^ The North West has the highest prevalence of alcohol- and diet-related risk factors for CLD in England.^4^ Integrated Diagnostics for Early Detection of Liver Disease (ID-LIVER) was a large, collaborative research project based in Greater Manchester, funded to unite universities, NHS trusts and small and large commercial partners. It addressed critical gaps around improving the detection of CLD at reversible stages, moving diagnostics and initial management to community-based care, and enabling needs-based diagnostics and interventions.^5^ One work-package focused on identifying people in the community at risk of CLD (‘proactive case-finding’), using digital search tools in routine primary care data. People with one or more risk factors for ARLD or MASLD were invited to a community liver assessment clinic (CLAC) with a hepatologist and hepatology nurse, held in an accessible location (local healthcare facilities or a mobile screening van).^6^ In another work-package, the investigators used machine-learning algorithms to develop a risk-prediction tool identifying cases with a high probability of significant fibrosis (hereafter ‘ID-LIVER-Machine Learning (ML)’).^7^ Uniform data were collected for all participants—those in the proactive case-finding population attending a CLAC and routine referrals attending a hepatology outpatient clinic—including liver stiffness measurement (LSM) using vibration-controlled transient elastography (VCTE). These data were used to train and validate the ID-LIVER-ML model, using LSM ≥8.0 kPa—indicating high risk of significant fibrosis—as a reference standard. The variables ID-LIVER-ML uses are aspartate aminotransferase, body-mass index, HbA1c, platelet count, triglycerides, alanine aminotransferase, high-density lipoprotein and alkaline phosphatase. These variables had the highest mean feature importance as determined by a ranking-based selection from 67 common socio-demographic and biochemical variables.^7^

In this study, we use data from these work-packages to evaluate the cost effectiveness of strategies incorporating one or both of proactive case-finding and enhanced risk-stratification (ID-LIVER-ML), compared with standard referral pathways.

## Methods

We developed a decision-analytic model to evaluate the benefits, harms and costs of different strategies to identify patients with significant CLD (defined as METAVIR stage F2 or higher, using liver stiffness of ≥8 kPa on transient elastography as a proxy measure; see below) in the community setting. The analysis conforms to the National Institute for Health and Care Excellence (NICE) reference case, adopting an NHS and personal social services perspective on costs.^8^ This report follows the Consolidated Health Economic Evaluation Reporting Standards (CHEERS) statement (see online supplemental eAppendix 1).^9^

10.1136/bmjph-2025-003480.supp1 Supplementary data

### Strategies evaluated

**Figure 1:**
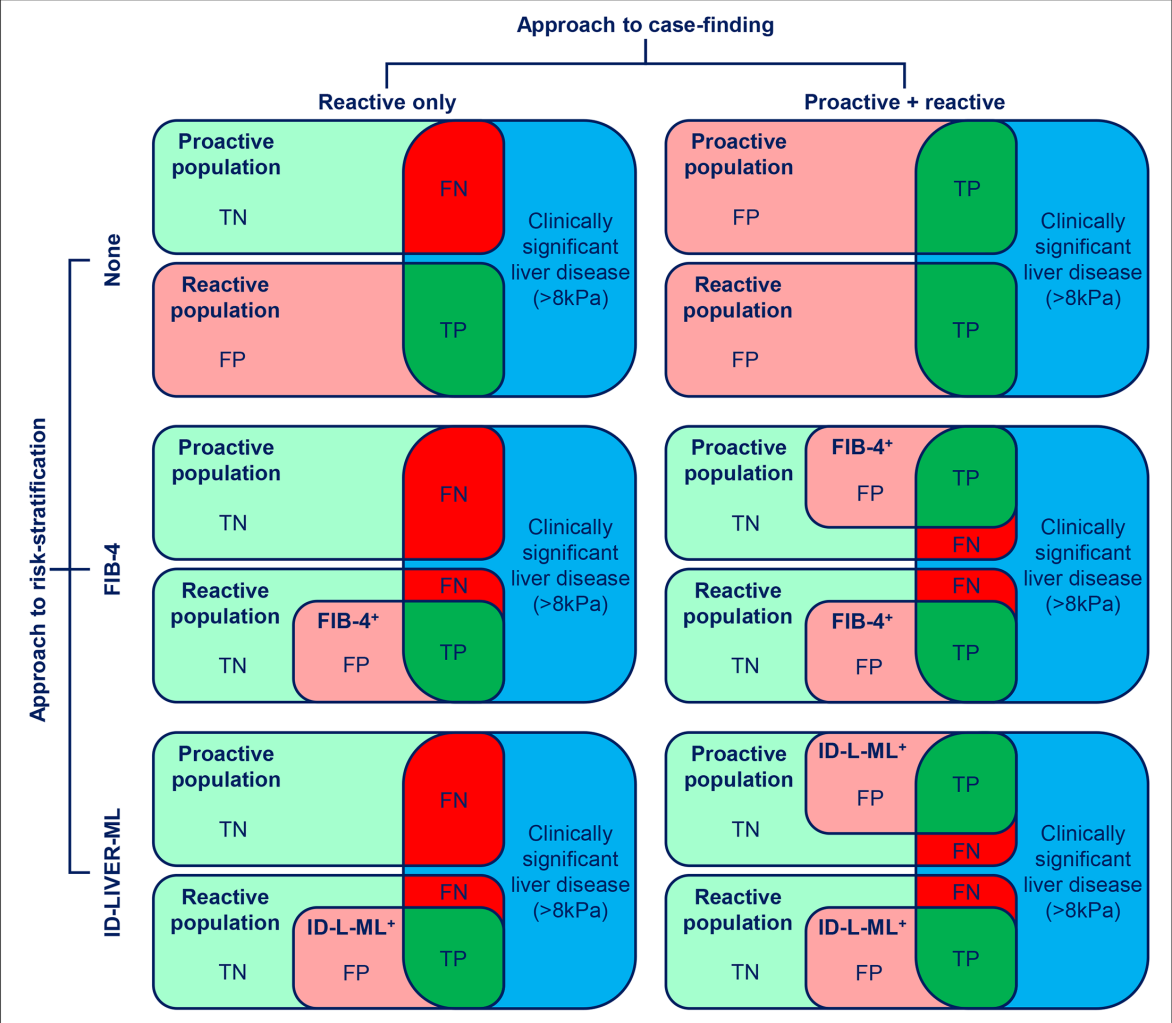
Illustration of the six strategies evaluated, combining two approaches to case-finding and three approaches to risk-stratification. FIB-4, Fibrosis-4 index; FN, false negative; FP, false positive; ID-L-ML, ID-LIVER machine-learning risk-prediction model; ID-LIVER, Integrated Diagnostics for Early Detection of Liver Disease; TN, true negative; TP, true positive.

We evaluate two approaches to case-finding and three approaches to risk-stratification, combining factorially to represent six possible strategies (figure 1). For case-finding, the ‘reactive’ approach represents the current pathway, where hepatology units passively receive referrals, primarily from general practitioners (GPs). The ‘reactive+proactive’ approach supplements routine referrals with case-finding in the community, as explored in ID-LIVER.^5^ The three approaches to risk-stratification are: none (everyone referred receives secondary care review); Fibrosis-4 (FIB-4) (limiting secondary care review to people with a given score) and ID-LIVER-ML (referral only for people with a given probability of significant CLD).

### Model structure

**Figure 2:**
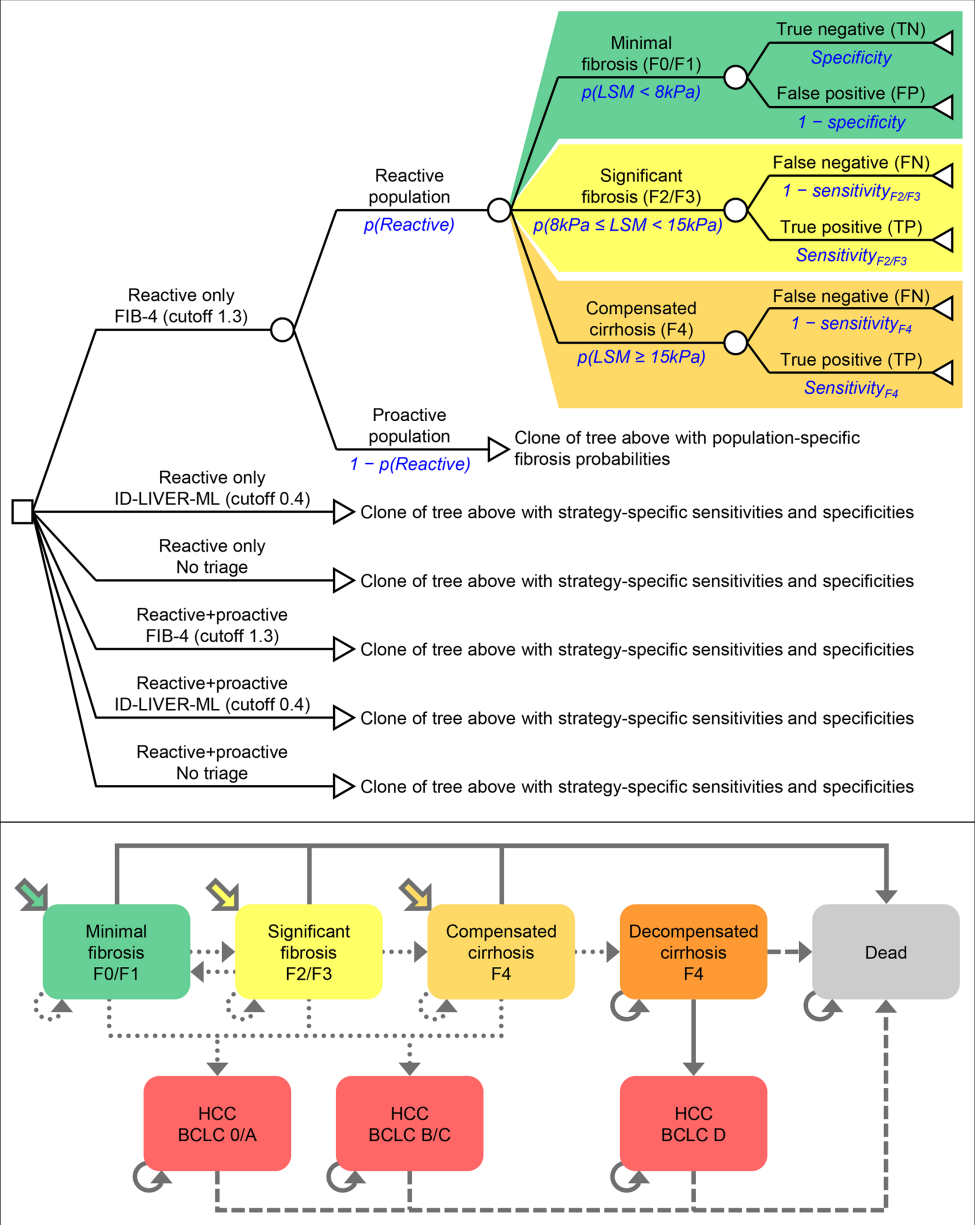
Model structure—decision-tree (identification probabilities) and Markov model (natural history). Upper: Decision-tree showing how the model apportions cohorts according to underlying level of fibrosis (minimal (F0/F1), significant (F2/F3), compensated cirrhosis (F4)) and whether the strategy in question identifies people in each category. Note that the model needs to simulate reactive and proactive populations for all strategies, regardless of whether the strategy in question targets one or both populations (meaning reactive-only strategies have sensitivity of 0 and specificity of 1 in the proactive population, classifying all people as either true- or false-negatives). Lower: Markov model simulating natural history of liver disease. Boxes show health-states. Arrows show possible transitions: dotted arrows show transitions that differ in probability between people with diagnosed and undiagnosed liver disease; dashed arrows show transitions where excess risk of death applies. Outlined block arrows identify states in which cohort initially enters model (as defined in terminal nodes of decision-tree). BCLC, Barcelona Clinic Liver Cancer staging system; F0/1/2/3/4, METAVIR fibrosis stage; FIB-4, Fibrosis-4 index; HCC, hepatocellular carcinoma; ID-LIVER-ML, ID-LIVER machine-learning risk-prediction model; ID-LIVER, Integrated Diagnostics for Early Detection of Liver Disease; LSM, liver stiffness measurement; p, probability.

We use two independent but structurally identical decision-analytic models—one each for ARLD and MASLD—and weight results together as a final step. The models begin with decision-trees that apportion the cohorts into three categories: minimal fibrosis (equivalent to METAVIR F0/F1), significant fibrosis (F2/F3) and compensated cirrhosis (F4), further subdividing according to the proportion in each category the strategy in question identifies or misidentifies (figure 2). Then, to simulate the natural and treated history of CLD, we use state-transition (Markov) models; figure 2 depicts their structure. Cohorts enter the state-transition models from the terminal nodes of the decision-tree. Subsequent states reflect long-term consequences of CLD: decompensated cirrhosis (note that this state does not necessarily reflect people experiencing acute decompensating events; rather it represents people who have had at least one such episode), hepatocellular carcinoma (HCC), and death. We use the Barcelona Clinic Liver Cancer staging system (BCLC)—a system commonly applied to prognostic evaluation and treatment planning of HCC^10^—to distinguish between early HCC (BCLC 0 /A), late HCC (BCLC B/C), and HCC arising in a failing liver (BCLC D). Decompensated cirrhosis and HCC are subject to excess, liver-specific mortality; other-cause deaths can occur in any state. Diagnosis of significant fibrosis leads clinicians to recommend lifestyle interventions that may slow disease progression. Thus, people correctly diagnosed have a lower probability of progressing to subsequent states, and may even reverse their liver damage. We assume that regression is not possible from cirrhosis. Once people enter the decompensated cirrhosis state, their CLD becomes known, regardless of whether it was previously diagnosed. People developing HCC come to attention 1 year later.

The models have annual cycles with half-cycle correction and a time horizon ensuring all cohorts reach a maximum age of 100. We apply a discount rate of 3.5% for costs and outcomes measured in quality-adjusted life-years (QALYs).

### Input parameters

Full details of all model input parameters appear in online supplemental eAppendix 2.

We base our simulated populations on data from ID-LIVER, comprising people with ARLD (n=286) and MASLD (n=850) identified via routine referral (n=840) or proactive case-finding (n=296). Starting age and sex depend on aetiology and stage of fibrosis; prevalence of each category of fibrosis depends on aetiology and mode of identification (online supplemental Table e1).

To simulate fibrosis progression in MASLD, we use a study reporting sequential biopsies.^11^ We derive analogous transition probabilities for ARLD from a multistate model we fitted to patient-level data available in three studies exploring fibrosis progression in liver biopsies (online supplemental eAppendix 3.1).^12–14^

Detecting CLD early leads to interventions that could slow or reverse disease progression. For MASLD, we use evidence from a UK study assessing the impact of a very-low-calorie diet.^15^ Our base-case uses 8-week follow-up data (immediately postintervention); in a scenario analysis, we explore the use of longer-term (9-month) outcomes from this study. Online supplemental eAppendix 3.2 explains parameter derivation. We assume uptake is as observed in recruitment for a trial assessing a similar intervention in people with diabetes.^16^ Effectiveness evidence for ARLD-specific behavioural interventions comes from three retrospective cohort studies reporting association with time to decompensation.^17–19^ In a scenario analysis, we use the effect of behavioural intervention plus medication from the same three studies. In both cases, uptake level comes from recruitment data for a UK-based trial in people with alcohol-use disorder (but not necessarily ARLD).^20^ In our base case, we assume that intervention effects influence all progression transitions in the model; in a scenario analysis, we explore the impact of limiting the effect to the individual transitions that most closely reflect the effectiveness evidence. A proportion of people with known compensated cirrhosis also benefits from HCC surveillance. We use UK evidence showing that the probability of finding HCC at an early stage is substantially higher in people undergoing surveillance, and project life expectancy accordingly.^21^ See online supplemental eAppendix 3.3 for details.

The effectiveness of ID-LIVER proactive case-finding is a simple function of the proportion of people with significant CLD successfully identified (ie, true-positives with LSM ≥8 kPa), balanced against the group whose liver health-check did not reveal cause for immediate concern (false-positives). For strategies in which no case-finding takes place, these proportions become false-negatives and true-negatives, respectively.

To estimate the diagnostic accuracy of FIB-4 and ID-LIVER-ML, we use the holdout dataset comprising 380 people from the ID-LIVER cohort whose data were not used in ID-LIVER-ML derivation. We split these individual data into fibrosis categories using LSM as a proxy measure: F0/F1 (LSM <8.0 kPa); F2/F3 (8.0 kPa≤LSM <15.0 kPa); F4 (15.0 kPa ≤LSM). We can then observe the number of people in each fibrosis category testing positive at any given threshold of FIB-4 and ID-LIVER-ML, and use these data to define probabilities for the decision-trees. For FIB-4, we use a cut-off of 1.3, as frequently cited.^22 23^ For ID-LIVER-ML, we start from the cut-off of 0.47 suggested by Purssell *et al*^7^ and then undertake threshold analysis to identify the cut-off that generates greatest net benefit. It is not possible to distinguish ARLD and MASLD in the holdout dataset, so we assume that risk-stratification tools work equally well across both populations.

Consistent with the study perspective, we account for resource-use associated with diagnosis and liver-related care only (online supplemental Table e5). This comprises four main elements: upfront costs (identification, risk-stratification and initial assessment); ongoing, fibrosis-stage-specific hepatology costs; lifestyle interventions for a proportion of diagnosed people and HCC treatment. All costs are in 2023/2024 British pounds, inflated where necessary using Personal Social Services Research Unit (PSSRU) inflators.^24^ Unit costs mostly derive from NHS Cost Collection and PSSRU.^24 25^ Online supplemental eAppendix 4 provides full details.

We account for primary care appointments and tests for reactive referrals. People assessed in secondary care incur costs of hepatologist time and transient elastography (with nurse time to deliver). We assume hepatology units can deliver these in a ‘one-stop’ clinic, rather than requiring a series of appointments, as we have shown in ID-LIVER.^6^ For proactive case-finding, we use estimates of £75 per person for identification (see online supplemental eAppendix 4.1.2.1) and £153 per person for CLAC attendance (online supplemental eAppendix 4.1.2.2). We assume that estimating FIB-4/ID-LIVER-ML incurs no cost (noting that we have already accounted for the blood tests that underpin them). We explore the possibility that ID-LIVER-ML may ultimately attract a licence fee in threshold analysis.

For ongoing hepatology care, we only account for liver-related resource use, so people with undiagnosed disease and those with F0/F1 fibrosis use no healthcare resources. We assume people with diagnosed F2/F3 fibrosis attend one hepatology outpatient appointment per year, including transient elastography and blood tests. For people with diagnosed compensated cirrhosis and anyone postdecompensation, we rely on costs estimated in NICE guideline NG50 (inflated to 2023/2024 values). We add the costs of twice-yearly ultrasound and biochemistry for a proportion of people undergoing HCC surveillance.

For the proportion of people with MASLD accepting lifestyle intervention, we account for dietitian time and use of meal-replacement products, directly reflecting the very low calorie diet we use for effectiveness estimates.^15^ For ARLD, we assume a 12-appointment behavioural intervention, as recommended by NICE guideline CG115. In our scenario analysis assuming cointervention with medication for alcohol use disorder, we include the cost of a 6-month course of naltrexone or acamprosate, also based on CG115. See online supplemental eAppendix 4.3 for details.

For HCC treatment, we mostly use costs estimated by Cullen *et al*,^26^ although a more complex calculation is required for people receiving systemic anticancer therapy; this relies heavily on NICE technology appraisal TA551. Online supplemental eAppendix 4.4 gives details.

Online supplemental Table S6 shows health-related quality of life (HRQoL) inputs; online supplemental eAppendix 5 provides details of derivation. We use EuroQol 5-dimensions 5-levels (EQ-5D-5L) measurements from the ID-LIVER cohort, crosswalked to EuroQol 5-dimensions 3-levels (EQ-5D-3L) values,^27^ to estimate HRQoL in people with compensated CLD. We translate absolute estimates into utility multipliers by dividing them by expected HRQoL for age-matched and sex-matched general population for each category.^28^ We calculate a pooled multiplier to reflect progression from compensated to decompensated cirrhosis from studies that report EQ-5D values for both states (online supplemental eAppendix 5.1). For HRQoL associated with HCC, we use evidence from a study examining the impact of early-stage and late-stage HCC diagnosis on HRQoL measured using 36-Item Short Form Survey (SF-36),^29^ which we map to EQ-5D values.^30^

### Implementation and sensitivity analysis

We built the model in Microsoft Excel. Probabilistic sensitivity analyses rely on 10 000 simulations, sampling all parameters from appropriate uncertainty distributions (mostly empirical; where unavailable, we assume SE 20% of mean; see online supplemental eAppendices 2–5). We use bootstrapping to account for uncertainty in diagnostic accuracy inputs, taking a random sample of the ID-LIVER holdout population with replacement for each iteration of the model. We performed deterministic (one-way) sensitivity analyses to explore individual parameters’ influence on model outputs and scenario analyses as noted above (see online supplemental eAppendix 12 for a list).

### Patient and public involvement

ID-LIVER had a collaborative approach to patient and public involvement throughout all stages of project design, development and delivery. This was led by Vocal (wearevocal.org), a not-for-profit health research organisation, and involved regular input from two patient governance advisors with lived experiences of CLD risk factors. They attended work package meetings and participated in a monthly forum with the research team. The team actively consulted underserved community groups with CLD risk factors to improve communication and participation using tailored resources.

## Results

### Natural history model

**Figure 3:**
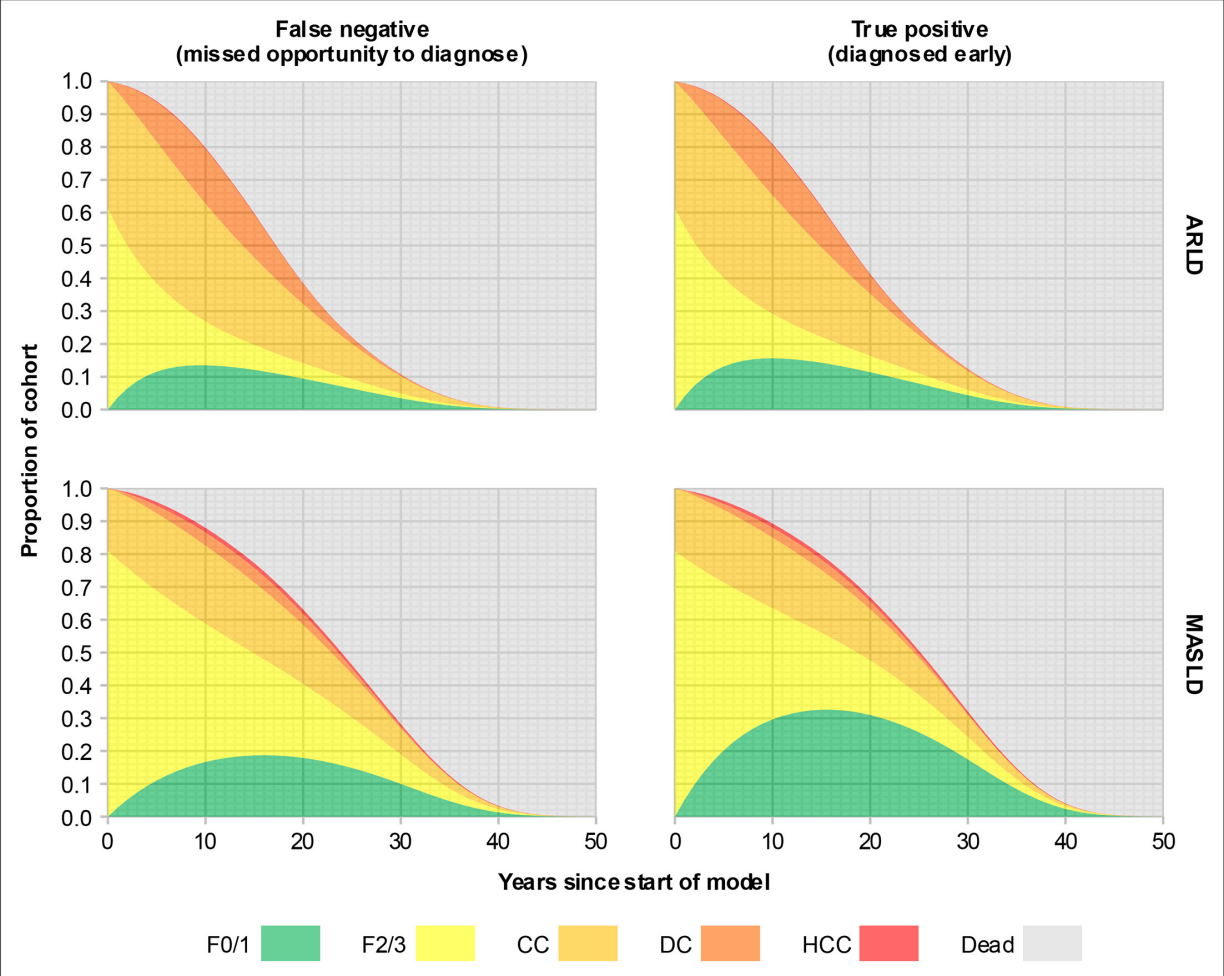
State occupancy over time for people with significant fibrosis, according to aetiology (ARLD vs MASLD) and diagnosis status (false-negative vs true-positive). ARLD, alcohol-related liver disease; CC, compensated cirrhosis; DC, decompensated cirrhosis; F0/1/2/3/4, METAVIR fibrosis stage; HCC, hepatocellular carcinoma; MASLD, metabolic dysfunction associated steatotic liver disease.

We start by estimating the impact early diagnosis of significant CLD—however achieved—has on expected lifetime costs and QALYs. Figure 3 shows the difference in prognosis for true-positives and false-negatives in ARLD and MASLD populations (online supplemental eAppendix 6 gives separate graphs for each fibrosis state). In MASLD, the ratio of people in the F2/F3 state as opposed to compensated cirrhosis at baseline is approximately 75:25, whereas it is closer to 50:50 for people with ARLD. The most obvious impact of successful detection is regression of disease severity from F2/F3 to F0/F1 (ie, the larger green area in true-positives), particularly in the MASLD population. There is also somewhat lower progression into the compensated cirrhosis, decompensated cirrhosis and HCC health states. The HCC health state is inconspicuous because relatively few people experience this event and most that do move quickly into the dead state. This is particularly true in ARLD, because incidence is lower and mortality associated with decompensated cirrhosis is higher in this population.

By aggregating costs and QALYs associated with predicted time in each state, we can estimate the impact of diagnosing versus not diagnosing CLD (see online supplemental Table e16, online supplemental eAppendix 7). We find that, if we value QALYs at £20 000 each (NICE-recommended cost-effectiveness threshold), any programme that detects significant CLD at a cost of less than £3300 per case would generate positive net benefit, compared with no detection. Using these data, we can estimate the cost-effectiveness of any risk-stratification strategy for which we know sensitivity, specificity and upfront costs (see online supplemental Figure e3, online supplemental eAppendix 7).

### Cost-effectiveness of case-finding and risk-stratification

**Figure 4:**
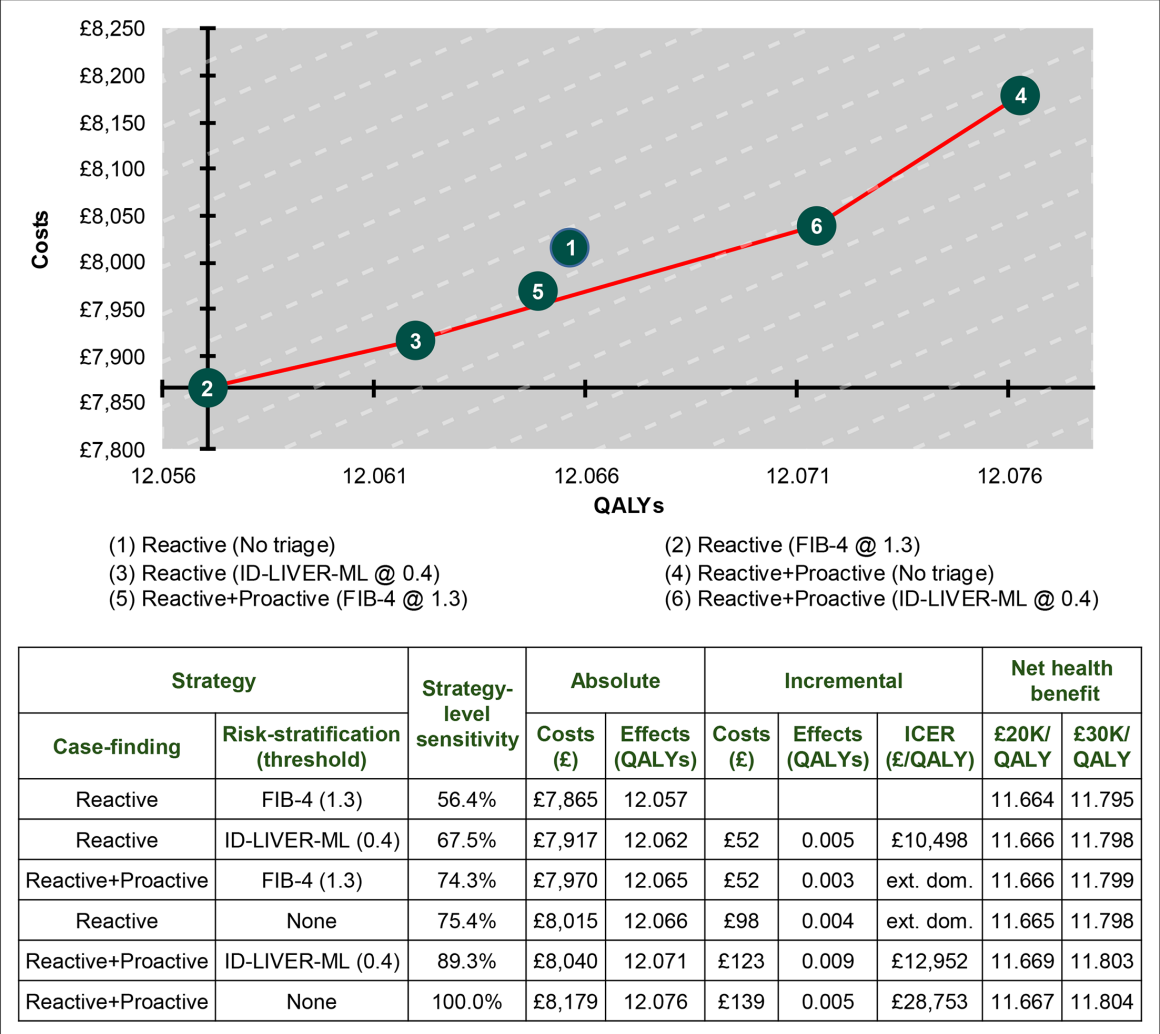
Cost-effectiveness of different approaches to case-finding and risk-stratification as assessed in ID-LIVER. Solid red line shows cost-effectiveness frontier. Dashed line in background shows iso-net-benefit gradient equal to £20 000 per QALY. If the line between any two points is shallower than this gradient, the option on the right (generating most QALYs) represents good value; if it is steeper, the option on the left is better value, assuming we value QALYs at £20 000 each. The subtable shows fully incremental analysis (ie, incremental values reflect the comparison between each option and the next-cheapest non-dominated alternative). Strategy-level sensitivity is the proportion of people with significant CLD recruited across reactive and proactive populations in ID-LIVER who would be identified by the strategy in question. All outcomes represent expected values per person within the whole at-risk population. All outcomes discounted at 3.5% per year. CLD, chronic liver disease; Ext. dom., extendedly dominated; FIB-4, Fibrosis-4 index; ICER, incremental cost-effectiveness ratio; ID-L-ML, ID-LIVER machine-learning risk-prediction model; ID-LIVER, Integrated Diagnostics for Early Detection of Liver Disease; QALY, quality-adjusted life-years.

Cost-effectiveness results for the six strategies our model simulates using the previously suggested ID-LIVER-ML cut-off of 0.47 are in online supplemental eAppendix 8. However, threshold analysis (online supplemental eAppendix 9) reveals that a cut-off of 0.4 generates greater net benefit, so this is the value we use for all further analyses. Figure 4 shows incremental cost-effectiveness results using the optimised cut-off. The cheapest strategy is FIB-4-based triage for reactive referrals, because it is the approach that classifies most people—rightly or wrongly—as negative. Switching to risk-stratification with ID-LIVER-ML in the same population identifies more true-positives, generating 0.005 extra QALYs per person at an incremental cost of £52 (~£10 500 per QALY gained). Extending the population to include proactive case-finding generates a further 0.009 QALYs, at a cost of £123 per person (~£13 000 per QALY gained). We would gain most QALYs by combining reactive and proactive populations and applying no risk-stratification (under which strategy, hepatologists will see 100% of people who might be at risk). However, this is also the most expensive strategy, and the incremental QALY-gain, compared with ID-LIVER-ML-based triage in the same population, would only be sufficient to counterbalance the incremental cost if we value QALYs at more than £28 000 each.

### Sensitivity analyses

Probabilistic sensitivity analysis outputs appear in online supplemental eAppendix 10. If we value QALYs at £20 000 each, the probability that some form of risk-stratification with ID-LIVER-ML is optimal is around 0.7, and the probability that combining it with proactive case-finding provides greatest net benefit is around 0.5. FIB-4-led strategies have less than 0.25 probability of providing best value.

One-way sensitivity analysis (online supplemental eAppendix 11) shows that we are fairly confident that ID-LIVER-ML is a better risk-stratification tool than FIB-4 in the reactive-only population (unless either lifestyle intervention is at the less effective bound of its 95% CI) and, if we extend to the reactive+proactive population, ID-LIVER-ML is better than no risk-stratification. It is somewhat more uncertain whether extending from reactive-only referrals to proactive case-finding represents good value for money; effectiveness estimates for lifestyle interventions are the most obviously influential parameters (explored further in threshold analysis, below).

Scenario analyses (online supplemental eAppendix 12) suggest that attenuating the benefit of diagnosis, by limiting the effect of lifestyle intervention to only one transition, means that the optimal strategy would be to see as few people as possible in secondary care (using FIB-4 in a reactive-only population). Conversely, diagnosis gets more valuable when we assume that HRQoL changes with fibrosis-stage; if we believe this, then it would probably be worth abandoning risk-stratification, and seeing everyone with risk factors in secondary care. Other scenario analyses (incorporating costs and effects of pharmacotherapy alongside behavioural intervention for ARLD; using 9-month follow-up evidence to estimate the long-term effectiveness of the MASLD dietary intervention; using an alternative approach to costing proactive case-finding) do not have a material effect on cost-effectiveness estimates. When we derive costs of proactive case-finding from the ID-LIVER research budget, overall results do not change materially, though the comparison between ID-LIVER-ML in reactive-only and proactive-and-reactive settings becomes more finely balanced.

Threshold analysis (online supplemental eAppendix 9) shows that lifestyle interventions would only have to be moderately less effective than our base-case estimate to make proactive case-finding bad value (OR ≥0.45 instead of 0.21 for MASLD; HR ≥0.90 instead of 0.70 for ARLD). When we apply a cost to the ID-LIVER-ML model, we find that it remains good value for money at any cost ≤£42 per person.

## Discussion

### Principal findings

Our study confirms the existence of a reservoir of people with undiagnosed CLD in the community and shows that we can find some of them in targeted ways that represent good value for money. We also demonstrate that we can improve risk-stratification, both of these proactively identified people and the reactive referrals that remain hepatology services’ stock-in-trade. Although FIB-4-based triage, as commonly used in the UK, is likely to reduce referrals and minimise secondary care costs, it does so at the expense of ruling out some people with significant CLD. Our analysis suggests that ID-LIVER-ML can minimise such false-negatives, increasing population health at an incremental cost that reflects an effective use of NHS resources.

### Strengths

Our study relies on a relatively large dataset, collected in a region with a particular need for diagnostic strategies that will identify people with significant CLD. It is an additional advantage that all participants received a test that is likely to classify their liver health accurately (transient elastography), in contrast to many other studies, in which people who are perceived to be at low risk receive no further investigation. We explicitly simulate the benefits of diagnosis, using best-available empirical data on the healthcare costs, health effects, and likely uptake of lifestyle interventions.

We are able to identify evidence-based, optimal thresholds for risk-stratification tools, based on expected lifetime population-level net benefit, which contrasts with the arbitrary cut-offs that are currently prevalent. For example, using 1.3 for FIB-4 is influenced by a US study that targeted a negative predictive value of 90% without any empirical foundation.^23^ We find that triaging cases using FIB-4 at a cut-off of 1.3 does more net harm than good, and this finding is independent of our exploration of ID-LIVER-ML. Of note, this result does not arise because FIB-4 performs worse in the ID-LIVER dataset than in others (sensitivity 0.745 (95% CI 0.597 to 0.861); specificity 0.679 (95% CI 0.628 to 0.728) in the holdout data; see online supplemental Table e3 in online supplemental eAppendix 2.2). However, a sensitivity of 75% implies missing 25% of people with significant CLD, and we find that this missed opportunity outweighs the additional cost of seeing everyone in secondary care.

Our sensitivity analyses enable us to stress-test the analysis and identify areas of greatest uncertainty; unsurprisingly, we find that the parameters with greatest influence on decision-uncertainty are those reflecting the extent to which lifestyle interventions change the prognosis of people whose CLD has been identified. Similarly, a wide range of scenario analyses allows us to assess how changes in our key assumptions impact the choice of the optimal case-identification strategy. Mostly, these confirm our base-case results, though we find that case-identification strategies that find more people with CLD would no longer provide good value if lifestyle interventions only affect single health-state transitions. Conversely, if change in fibrosis state directly affects quality of life, we conclude that the optimal strategy is to refer all people with risk factors to secondary care.

### Limitations

All diagnostic accuracy estimates in our study use VCTE as a proxy for true liver pathology. Investigators have shown high concordance between LSM and histology in both ARLD^31–33^ and MASLD,^34^ but it is inevitable that some of the true-positives/false-negatives in our calculations do not have meaningful fibrosis and some of the true-negatives/false-positives do. This may be a particular problem in people with high BMI: Myers *et al* found that discordance in fibrosis staging was four to five times more frequent in patients with class III obesity (BMI ≥40 kg/m^2^) and liver stiffness >7 kPa.^35^ However, for practical purposes, the distinction is moot: liver biopsy is becoming ever-rarer and is, itself, an imperfect test that may misclassify people.^36^ Therefore, we believe LSM represents as accurate an indicator of fibrosis-stage as is viable in present-day practice.

Our model simulates a one-time opportunity for identification, assuming that any case that is missed will only come to light when symptoms develop. On the one hand, this assumption may overstate value of case-finding, as people may still receive a diagnosis while asymptomatic via later healthcare contacts. On the other, it may understate value of case-finding, as people who are seen but—correctly or incorrectly—discharged may still benefit from lifestyle advice and may also be primed to heed future symptoms.

We had to model the natural history of undiagnosed disease, which, by definition, is unobservable. The sequential biopsy studies we use probably provide a reasonable proxy, though any lifestyle modification participants adopted between biopsies may serve to bias true natural progression rates downwards. In this respect, it may not be a bad thing that our ARLD evidence^12–14^ comes from a historical era (1970s–1980s), during which behaviour-modification interventions were unusual. However, the studies also predate modern-day histopathological criteria, meaning we had to approximate the relationship between reported fibrosis findings and modelled states. We acknowledge that this introduced uncertainty to the analysis, but the direction and magnitude of any bias are uncertain.

We do not simulate any pharmacological treatments that may slow progression of CLD. NICE’s cirrhosis guideline (NG50) gives a weak (‘consider’) recommendation for beta-blockers for primary prevention of decompensation, based largely on a Spanish randomised trial.^37^ Previous cost-effectiveness analyses^38 39^ have assumed people with diagnosed CLD gain benefit from pioglitazone; there is some evidence this may be true,^40^ though we believe it is uncommonly prescribed for this indication in the UK (and is off-label when it is). There is emerging evidence that GLP-1 receptor agonists may reduce LSMs and/or fibrosis.^41 42^ Although none has a marketing authorisation explicitly covering MASLD, NICE currently recommends semaglutide for people with high BMI and ‘at least 1 weight-related comorbidity’ (TA875), as which MASLD would qualify. If any of these agents—or MASLD-specific medicines such as resmetirom^43^ which, at the time of writing, has recently received EMA approval and will imminently undergo NICE appraisal in the UK—provide benefit at acceptable cost, and hepatologists enable access to them for at least some people with significant fibrosis, our analysis will underestimate the value for money with which each true-positive diagnosis is associated.

Our analysis inherits any shortcomings in the evidence we use to quantify the extent to which lifestyle intervention impacts fibrosis progression. For both ARLD and MASLD, these effectiveness parameters come from non-randomised evidence, which may give biased estimates of effect. To mitigate this risk, we undertook extensive sensitivity analysis around all relevant parameters. For MASLD, we explored 9-month effects from our chosen source instead of the 8-week estimate we use in our base case. For ARLD, we used effects with or without pharmacological cointervention. In both cases, results were not meaningfully different from our base case. We also varied both effectiveness parameters across wide ranges and varied our assumption that effects apply across all fibrosis-progression parameters. These analyses reach the predictable conclusion that, if hepatologists have less ability to influence disease progression than we like to think, case-identification strategies that minimise costs and find fewer people would quickly become optimal. However, there are factors that may counterbalance any nihilism this finding induces. In particular, our model focuses solely on liver outcomes, so is unable to capture any other benefits. People with ARLD or MASLD for whom behaviour modification is effective are also likely to benefit from reduced cardiovascular risk, improved mental health, and a reduction in cancer risk that extends beyond the liver.^44–46^ In this way, our analysis almost certainly underestimates the benefit of diagnosing and referring people to effective lifestyle modifications in both populations.

Our model of advanced liver disease is necessarily simplified. For example, we recognise that some patients who have had a decompensating event may consume fewer resources (perhaps after benefiting from lifestyle modifications that may induce disease regression), whereas others experience recurrent decompensating events leading to repeated inpatient episodes and higher healthcare use. The epidemiological data that underpin our model comprise a mix of people fitting these profiles and all points in between. As it is not possible to separate them, the numbers used represent the average resource use of a person who has experienced one or more decompensating event. This will produce valid results as long as the populations in the underlying data had a similar prognostic case-mix to the people in our decision-problem.

The ID-LIVER-ML model was derived and validated in a mixed population of people from Nottingham and Manchester. The model shows good validity across these two populations,^7^ and all accuracy parameters in this paper come from the holdout set that was not used in model derivation. Nevertheless, independent, external validation of the tool would enhance confidence in our findings. However, we would also emphasise that our other results are independent of ID-LIVER-ML. In particular, we find that proactive case-finding in the community provides good value for money whether or not any particular triage tool is used. Moreover, we do not only find that triage with FIB-4 is less cost-effective than ID-LIVER-ML; we find that it is inferior to a ‘no triage’ strategy where all people at risk of CLD receive secondary care assessment.

### Comparison with other research

There are three UK-focused analyses assessing referral pathways for people with suspected MASLD. Srivastava *et al* assessed multiple risk-stratification algorithms relying on non-invasive tests including FIB-4, Enhanced Liver Fibrosis (ELF) and elastography for people in primary care with raised liver enzymes.^47^ The authors find that non-invasive tests could increase true-positive referrals to secondary care while reducing false-positives, compared with unassisted GP decision-making (although their estimates of the accuracy of the latter are not based on any empirical data). They also find that identifying people with significant CLD yields net cost-savings when accounting for prevention of downstream events. This finding contrasts with ours; however, there are multiple differences in methods and inputs. Critically, we specify, evidence, and account for the costs of a mechanism (lifestyle intervention) by which diagnosis may slow disease progression, where Srivastava *et al* assume benefits without intervention costs. We also note that unit-costs of hepatology appointments have doubled in the time since their analysis. Finally, we note that Srivastava *et al*’s preferred pathway excludes all people with FIB-4 <1.3; our findings suggest that around 25% of such people have significant CLD, and strategies that do not automatically rule them out generate better value.

Tanajewski *et al* model a community risk-stratification pathway using transient elastography to assess people at risk of MASLD.^39^ They find a 0.85 probability that the approach is associated with an ICER of £20 000 per QALY or better, compared with standard care. A shortcoming of the model is that, as limited evidence was available at the time, it quantifies the benefit of diagnosis by assuming 100% of people receive pharmacological treatment with pioglitazone, although this is uncommon in practice. Moreover, the effect-measure comes from a trial using a different drug (rosiglitazone) that has been withdrawn from the UK market.

Crossan *et al* assess non-invasive tests to triage secondary care referrals for people with MASLD.^48^ They find that all approaches deliver cost-savings, compared with indiscriminate referrals. They do not model consequences of correct/incorrect diagnoses (eg, through expected lifetime QALYs). However, they suggest that greatest net benefit, across a range of hypothesised values indicating the relative importance of true-positives and false-positives, comes from a two-tiered approach using FIB-4 followed by ELF in indeterminate cases.

There are no cost-effectiveness analyses focusing on identifying ARLD in the UK. Asphaug *et al* simulate various approaches to screening for ARLD in Denmark.^49^ They model a hypothetical impact of diagnosis on drinking behaviour, assuming that CLD will only progress in people who continue to drink. They find that risk-stratification with ELF followed by elastography for positive cases is likely to be optimal in primary care, but an elastography-only approach is better in secondary care (although it is unclear why the elastography-only pathway generates fewer QALYs in primary care, when it has most true-positives in both scenarios).

We are not aware of any previous cost-effectiveness analyses looking at proactive case-finding in a broad community at risk for CLD, although one UK-focused cost-effectiveness analysis suggests it would be good value to screen for MASLD in people with type 2 diabetes.^38^

### Conclusions

Our economic evaluation suggests that early detection of ARLD and MASLD has the anticipated benefits for the person and can be achieved in ways that represent good value for the system. Our conclusions depend importantly on the effectiveness of interventions to delay progression of CLD. However, any overestimate, in this domain, may be offset by other benefits that we cannot quantify (informal lifestyle advice; possible pharmacological interventions; spillover effects of liver-motivated behaviour-change for non-hepatic outcomes). We continue to undertake proactive case-finding in Greater Manchester, collecting similar data as in ID-LIVER. We intend to use these data to explore potential refinements to the approaches evaluated here. In particular, we aim to improve the specificity of initial digital searches in primary care records, assessing which (combinations of) high-level risk factors identify people at greatest risk of significant CLD.

## Data Availability

Data may be obtained from a third party and are not publicly available. All data relevant to the study are included in the article or uploaded as supplementary information. This analysis predominantly relies on data that have been published elsewhere. We also use some patient-level data from ID-LIVER; see Purssell *et al* (https://doi.org/10.64898/2026.03.04.26347631) for the relevant data availability statement.
