# Supplementary material for: Proactive case-finding and risk-stratification in people at risk of chronic liver disease in Greater Manchester: a cost-effectiveness analysis
Source: BMJ Public Health. 2026 Jul 13;4(3):e003480. doi: 10.1136/bmjph-2025-003480 (PMC13365752; doi:10.1136/bmjph-2025-003480)
Supplement: Supplementary data [file bmjph-4-3-s001.pdf]

Proactive case-finding and risk-stratification in people at risk of chronic liver disease in Greater Manchester: a cost-effectiveness analysis

Gabriel Rogers[1*], Stephanie Landi[2,3], Huw Purssell[2,3], Tonia Momoh[1], Sol Yates[1], Oliver Street[2], Karen Piper Hanley[2], Neil Hanley[2,4,5], Varinder Athwal[2,3], Katherine Payne[1]

[1] Manchester Centre for Health Economics, Faculty of Medicine, Biology and Health, University of Manchester
[2] Faculty of Medicine, Biology and Health, University of Manchester
[3] Manchester University NHS Foundation Trust
[4] College of Medicine and Health, University of Birmingham
[5] University Hospitals Birmingham NHS Foundation Trust

[*] Correspondence to gabriel.rogers@manchester.ac.uk

BMJ Public Health 2026

http://dx.doi.org/10.1136/bmjph-2025-003480

SUPPLEMENTARY MATERIAL

eAppendix 1 CHEERS checklist 2
eAppendix 2 Model input parameters 4
eAppendix 3 Derivation of model parameters – natural history and intervention effectiveness 8
eAppendix 4 Derivation of model parameters – costs 12
eAppendix 5 Derivation of model parameters – health-related quality of life 21
eAppendix 6 State occupancy graphs for each starting state 24
eAppendix 7 Cost effectiveness of hypothetical strategies for identifying people with liver disease 25
eAppendix 8 Cost-effectiveness results using the previously suggested ID-LIVER-ML threshold of 0.47. 28
eAppendix 9 Threshold analyses 29
eAppendix 10 Probabilistic sensitivity analysis 33
eAppendix 11 One-way sensitivity analysis 35
eAppendix 12 Scenario analyses 38
References for eAppendices 46

eAppendix 1 CHEERS checklist

	Item	Guidance for Reporting	Reported on page
**TITLE**			
Title	1	Identify the study as an economic evaluation and specify the interventions being compared.	1
**ABSTRACT**			
Abstract	2	Provide a structured summary that highlights context, key methods, results and alternative analyses.	2
**INTRODUCTION**			
Background and objectives	3	Give the context for the study, the study question and its practical relevance for decision making in policy or practice.	4
**METHODS**			
Health economic analysis plan	4	Indicate whether a health economic analysis plan was developed and where available.	N/A
Study population	5	Describe characteristics of the study population (such as age range, demographics, socioeconomic, or clinical characteristics).	7
Setting and location	6	Provide relevant contextual information that may influence findings.	4
Comparators	7	Describe the interventions or strategies being compared and why chosen.	6
Perspective	8	State the perspective(s) adopted by the study and why chosen.	6
Time horizon	9	State the time horizon for the study and why appropriate.	6
Discount rate	10	Report the discount rate(s) and reason chosen.	6
Selection of outcomes	11	Describe what outcomes were used as the measure(s) of benefit(s) and harm(s).	6
Measurement of outcomes	12	Describe how outcomes used to capture benefit(s) and harm(s) were measured.	6
Valuation of outcomes	13	Describe the population and methods used to measure and value outcomes.	9
Measurement and valuation of resources and costs	14	Describe how costs were valued.	8
Currency, price date, and conversion	15	Report the dates of the estimated resource quantities and unit costs, plus the currency and year of conversion.	8
Rationale and description of model	16	If modelling is used, describe in detail and why used. Report if the model is publicly available and where it can be accessed.	6
Analytics and assumptions	17	Describe any methods for analysing or statistically transforming data, any extrapolation methods, and approaches for validating any model used.	7
Characterizing heterogeneity	18	Describe any methods used for estimating how the results of the study vary for sub-groups.	N/A
Characterizing distributional effects	19	Describe how impacts are distributed across different individuals or adjustments made to reflect priority populations.	N/A
Characterizing uncertainty	20	Describe methods to characterize any sources of uncertainty in the analysis.	10
Approach to engagement with patients and others affected by the study	21	Describe any approaches to engage patients or service recipients, the general public, communities, or stakeholders (e.g., clinicians or payers) in the design of the study.	10
**RESULTS**			
Study parameters	22	Report all analytic inputs (e.g., values, ranges, references) including uncertainty or distributional assumptions.	11
Summary of main results	23	Report the mean values for the main categories of costs and outcomes of interest and summarise them in the most appropriate overall measure.	11
Effect of uncertainty	24	Describe how uncertainty about analytic judgments, inputs, or projections affect findings. Report the effect of choice of discount rate and time horizon, if applicable.	12
Effect of engagement with patients and others affected by the study	25	Report on any difference patient/service recipient, general public, community, or stakeholder involvement made to the approach or findings of the study	10
**DISCUSSION**			
Study findings, limitations, generalizability, and current knowledge	26	Report key findings, limitations, ethical or equity considerations not captured, and how these could impact patients, policy, or practice.	14

	Item	Guidance for Reporting	Reported on page
**OTHER RELEVANT INFORMATION**			
Source of funding	27	Describe how the study was funded and any role of the funder in the identification, design, conduct, and reporting of the analysis	20
Conflicts of interest	28	Report authors conflicts of interest according to journal or International Committee of Medical Journal Editors requirements.	20

Husereau D, Drummond M, Augustovski F, de Bekker-Grob E, Briggs AH, Carswell C, Caulley L, Chaiyakunapruk N, Greenberg D, Loder E, Mauskopf J, Mullins CD, Petrou S, Pwu RF, Staniszewska S; CHEERS 2022 ISPOR Good Research Practices Task Force. Consolidated Health Economic Evaluation Reporting Standards 2022 (CHEERS 2022) Statement: Updated Reporting Guidance for Health Economic Evaluations. BMJ. 2022;376:e067975.

The checklist is Open Access distributed in accordance with the terms of the Creative Commons Attribution (CC BY 4.0) license, which permits others to distribute, remix, adapt and build upon this work, for commercial use, provided the original work is properly cited. See: http://creativecommons.org/licenses/by/4.0/.

eAppendix 2 Model input parameters

2.1. Baseline population

Table e1 Model parameters – baseline population

Parameter	Value (95% CI)	Probabilistic parameters	Source
Starting age (years)			
ARLD F0/1	55.4 (53.7, 57.1)	Normal: μ=55.40; σ=0.89	ID-LIVER cohort
ARLD F2/3	58.1 (55.4, 60.8)	Normal: μ=58.11; σ=1.37	
ARLD F4	58.6 (55.5, 61.8)	Normal: μ=58.63; σ=1.62	
MASLD F0/1	54.4 (53.3, 55.5)	Normal: μ=54.41; σ=0.55	
MASLD F2/3	57.1 (54.9, 59.2)	Normal: μ=57.05; σ=1.12	
MASLD F4	59.9 (55.8, 64.0)	Normal: μ=59.87; σ=2.09	
Sex (%male)			
ARLD F0/1	0.665 (0.595, 0.731)	Beta: α=121; β=61	ID-LIVER cohort
ARLD F2/3	0.557 (0.432, 0.679)	Beta: α=34; β=27	
ARLD F4	0.744 (0.605, 0.861)	Beta: α=32; β=11	
MASLD F0/1	0.452 (0.415, 0.489)	Beta: α=318; β=386	
MASLD F2/3	0.509 (0.418, 0.599)	Beta: α=59; β=57	
MASLD F4	0.600 (0.423, 0.765)	Beta: α=18; β=12	
Proportion of cases reactive			
ARLD	0.557 (0.509, 0.604)	Beta: α=235; β=187	ID-LIVER cohort (financial yr 2022/23)
MASLD	0.631 (0.546, 0.711)	Beta: α=82; β=48	
Proportion of cases with MASLD			
Reactive	0.731 (0.700, 0.760)	Beta: α=614; β=226	ID-LIVER cohort
Proactive	0.797 (0.750, 0.841)	Beta: α=236; β=60	
Weighted average	0.760		
Proportion with fibrosis at baseline			
Reactive			
ARLD			
F0/1 (LSM < 8kPa)	0.588 (0.524, 0.652)	Dirichlet: α_1=133; α_2=52; α_3=41	ID-LIVER cohort
F2/3 (8kPa ≤ LSM < 15kPa)	0.230 (0.178, 0.287)		
F4 (15kPa ≤ LSM)	0.181 (0.134, 0.234)		
MASLD			
F0/1 (LSM < 8kPa)	0.796 (0.764, 0.827)	Dirichlet: α_1=489; α_2=97; α_3=28	ID-LIVER cohort
F2/3 (8kPa ≤ LSM < 15kPa)	0.158 (0.130, 0.188)		
F4 (15kPa ≤ LSM)	0.046 (0.031, 0.063)		
Proactive			
ARLD			
F0/1 (LSM < 8kPa)	0.817 (0.710, 0.903)	Dirichlet: α_1=49; α_2=9; α_3=2	ID-LIVER cohort
F2/3 (8kPa ≤ LSM < 15kPa)	0.150 (0.072, 0.250)		
F4 (15kPa ≤ LSM)	0.033 (0.004, 0.091)		
MASLD			
F0/1 (LSM < 8kPa)	0.911 (0.872, 0.944)	Dirichlet: α_1=215; α_2=19; α_3=2	ID-LIVER cohort
F2/3 (8kPa ≤ LSM < 15kPa)	0.081 (0.049, 0.118)		
F4 (15kPa ≤ LSM)	0.008 (0.001, 0.023)		

ARLD = alcohol-related liver disease; F0/1/2/3/4 = METAVIR fibrosis stage; LSM = liver stiffness measurement; MASLD = metabolic-dysfunction-associated steatotic liver disease

Rogers G, et al. BMJPH 2026; 4:e003480. doi: 10.1136/bmjph-2025-003480

2.2. Natural history, accuracy of risk-stratification tools, and effectiveness of interventions

Table e2 Model parameters – natural history

Parameter	Value (95% CI)	Probabilistic parameters	Source
Annual probability of fibrosis change			
ARLD			
F0/1→F0/1	0.946 (0.907, 0.978)	Bootstrapped output of multistate model; see *eAppendix2.1*	[1–3]
F0/1→F2/3	0.051 (0.021, 0.089)		
F0/1→CC	0.003 (0.001, 0.005)		
F2/3→F0/1	0.059 (0.021, 0.107)		
F2/3→F2/3	0.846 (0.774, 0.906)		
F2/3→CC	0.094 (0.049, 0.151)		
MASLD			
F0/1→F0/1	0.965 (0.955, 0.974)	Dirichlet (7.21yr): α_1=164; α_2=48	[4]
F0/1→F2/3	0.035 (0.026, 0.045)		
F2/3→F0/1	0.034 (0.022, 0.049)	Dirichlet (7.21yr): α_1=24; α_2=65; α_3=20	
F2/3→F2/3	0.938 (0.910, 0.961)		
F2/3→CC	0.028 (0.017, 0.041)		
CC → DC rate per year			
ARLD	0.073 (0.065, 0.082)	Lognorm: μ=−2.62; σ=0.06	[5]
MASLD	0.027 (0.016, 0.044)	Lognorm: μ=−3.62; σ=0.25	[6]
HCC rate per year			
ARLD no cirrhosis	0.000005	–	Assume proportional
ARLD with cirrhosis	0.0032 (0.0021, 0.0048)	Lognorm: μ=−5.759; σ=0.213	[7]
MASLD no cirrhosis	0.00005 (0.00004, 0.00006)	Lognorm: μ=−10.01; σ=0.11	[8]
MASLD with cirrhosis	0.023 (0.020, 0.026)	Lognorm: μ=−3.785; σ=0.066	[9–11]
Excess mortality: DC -v- gen. pop.			
ARLD SMR year 1	17.7 (14.5, 21.6)	Lognorm: μ=2.87; σ=0.10	[12]
ARLD SMR year 2 onwards	11.0 (9.4, 12.8)	Lognorm: μ=2.40; σ=0.08	
MASLD SMR year 1	13.70 (11.51, 16.31)	Lognorm: μ=2.62; σ=0.09	
MASLD SMR year 2 onwards	5.20 (4.38, 6.17)	Lognorm: μ=1.65; σ=0.09	
HCC survival (exponential)			
Ln(rate)	−1.80 (−1.96, −1.63)	Multivariate normal; see *eAppendix3.3*	[13]
Ln(HR) BCLC B/C -v- 0/A	1.36 (1.17, 1.54)		
Ln(HR) BCLC D -v- 0/A	2.44 (2.22, 2.66)		

ARLD = alcohol-related liver disease; BCLC = Barcelona Clinic Liver Cancer stage; CC = compensated cirrhosis; DC = decompensated cirrhosis; F0/1/2/3/4 = METAVIR fibrosis stage; gen. pop. = general population; HCC = hepatocellular carcinoma; lognorm = lognormal; MASLD = metabolic-dysfunction-associated steatotic liver disease; SMR = standardised mortality ratio

Table e3 Model parameters – accuracy of risk-stratification tools

Parameter	Value (95% CI)	Probabilistic parameters	Source
Accuracy of risk-stratification tools			
Specificity (LSM < 8kPa)			ID-LIVER holdout set
No risk-stratification	0.000	–	
FIB-4 (1.3 threshold)	0.679 (0.628, 0.728)	Beta: α=226; β=107	
ID-LIVER-ML (0.4 threshold)	0.562 (0.508, 0.614)	Beta: α=187; β=146	
Sensitivity (8kPa ≤ LSM < 15kPa)			
No risk-stratification	1.000	–	
FIB-4 (1.3 threshold)	0.714 (0.556, 0.849)	Beta: α=25; β=10	
ID-LIVER-ML (0.4 threshold)	0.886 (0.763, 0.967)	Beta: α=31; β=4	
Sensitivity (15kPa ≤ LSM)			
No risk-stratification	1.000	–	
FIB-4 (1.3 threshold)	0.833 (0.587, 0.977)	Beta: α=10; β=2	
ID-LIVER-ML (0.4 threshold)	0.917 (0.715, 0.998)	Beta: α=11; β=1	
Sensitivity (8kPa ≤ LSM)			Not directly used in model, but provided for comparability
No risk-stratification	1.000	–	
FIB-4 (1.3 threshold)	0.745 (0.597, 0.861)	Beta: α=35; β=12	
ID-LIVER-ML (0.4 threshold)	0.894 (0.769, 0.965)	Beta: α=42; β=5	

LSM = liver stiffness measurement

Table e4 Model parameters – effectiveness of interventions

Parameter	Value (95% CI)	Probabilistic parameters	Source
Effectiveness: lifestyle intervention			
MASLD			
OR for progression	0.212 (0.081, 0.555)	Lognorm: μ=−1.55; σ=0.49	[14]
Uptake of intervention	28.0% (25.8%, 30.3%)	Beta: α=423; β=1087	[15]
ARLD			
HR for progression	0.697 (0.479, 1.015)	Lognorm: μ=−0.36; σ=0.19	[16–18]
Uptake of intervention	29.3% (24.2%, 34.7%)	Beta: α=84; β=203	[19]
Effectiveness: HCC surveillance			
Prob of BCLC 0/A,no surveillance	0.177 (0.143, 0.214)	Beta: α=78; β=362	[20]
OR for BCLC 0/A, surv. -v- none	4.511 (3.271, 6.222)	Lognorm: μ=1.51; σ=0.16	
Surveillance uptake	67.7% (63.1%, 72.2%)	Beta: α=277; β=132	[13]

ARLD = alcohol-related liver disease; BCLC = Barcelona Clinic Liver Cancer stage; HCC = hepatocellular carcinoma; HR = hazard ratio; lognorm = lognormal; MASLD = metabolic-dysfunction-associated steatotic liver disease; OR = odds ratio

2.3. Costs and quality of life

Table e5 Model parameters – summary of costs

Parameter	Value (95% CI)	Probabilistic parameters	Source
Costs			
Reactive referral (1° care)	£181 (£117, £259)	Gamma: α=25.00; β=7.26	See *eAppendix 4.1.1*
Reactive referral (2° care)	£296 (£191, £422)	Gamma: α=25.00; β=11.83	
Proactive case-finding (1° care)	£75 (£48, £107)	Gamma: α=25.00; β=2.20	See *eAppendix 4.1.2*
Proactive case-finding (2° care)	£153 (£99, £219)	Gamma: α=25.00; β=6.13	
Lifestyle intervention for MASLD	£1,208 (£782, £1,725)	Gamma: α=25.00; β=48.31	See *eAppendix 4.3*
Lifestyle intervention for ARLD	£862 (£558, £1,231)	Gamma: α=25.00; β=34.46	
Annual state-specific costs			
Undiagnosed liver disease	£0	See *eAppendix 4*	Various, but particular reliance on NICE NG50, Cullen et al. (2023)[21], National cost collection 2023/24[22], and PSSRU 2023/24[23] See *eAppendix 4* for full details.
F0/1 known	£0		
F2/3 known	£309.96		
CC known	£475.71		
CC known + HCC surveillance	£596.85		
DC (MASLD)	£16,612.81		
DC (ARLD)	£24,169.21		
HCC BCLC 0/A Yr1	£15,018.93		
HCC BCLC 0/A Yr2	£15,128.52		
HCC BCLC 0/A Yr3+	£4,801.62		
HCC BCLC B/C Yr1	£12,639.41		
HCC BCLC B/C Yr2	£13,008.73		
HCC BCLC B/C Yr3+	£4,801.62		
HCC BCLC D Yr1+	£21,739.80		

ARLD = alcohol-related liver disease; BCLC = Barcelona Clinic Liver Cancer stage; CC = compensated cirrhosis; DC = decompensated cirrhosis; F0/1/2/3/4 = METAVIR fibrosis stage; HCC = hepatocellular carcinoma; MASLD = metabolic-dysfunction-associated steatotic liver disease

Table e6 Model parameters – quality of life

Parameter	Value (95% CI)	Probabilistic parameters	Source
Quality of life: health-state utilities			
MASLD F0/1	0.773 (0.753, 0.793)	Normal: μ=0.773; σ=0.010	ID-LIVER cohort
Age- and sex-matched gen. pop.	0.855	See *eAppendix 5.1*	[24]
Multiplier -v- gen. pop.	0.904		
MASLD F2/3/4	0.658 (0.606, 0.710)	Normal: μ=0.658; σ=0.026	ID-LIVER cohort
Age- and sex-matched gen. pop.	0.845	See *eAppendix 5.1*	[24]
Multiplier -v- gen. pop.	0.779		
ARLD F0/1	0.750 (0.711, 0.790)	Normal: μ=0.750; σ=0.020	ID-LIVER cohort
Age- and sex-matched gen. pop.	0.857	See *eAppendix 5.1*	[24]
Multiplier -v- gen. pop.	0.876		
ARLD F2/3/4	0.667 (0.606, 0.728)	Normal: μ=0.667; σ=0.031	ID-LIVER cohort
Age- and sex-matched gen. pop.	0.847	See *eAppendix 5.1*	[24]
Multiplier -v- gen. pop.	0.787		
Multiplier, DC -v- other states	0.879 (0.824, 0.939)	Lognorm: μ=−0.128; σ=0.033	See *eAppendix 5.2*
Multiplier, early HCC -v- none	0.982	See *eAppendix 5.3*	[25]
Multiplier, late HCC -v- none	0.880		

ARLD = alcohol-related liver disease; DC = decompensated cirrhosis; F0/1/2/3/4 = METAVIR fibrosis stage; gen. pop. = general population; HCC = hepatocellular carcinoma; lognorm = lognormal; MASLD = metabolic-dysfunction-associated steatotic liver disease

eAppendix 3 Derivation of model parameters – natural history and intervention effectiveness

3.1. Derivation of transition probabilities for ARLD progression

To parameterise our model, we needed to identify studies representing natural progression of fibrosis in ARLD, which we could use to simulate the natural history of liver disease. Parker et al. (2019)[26] systematically reviewed studies reporting histological progression in people with ARLD. They do not report their results in a way that is useful for our purposes, but we reviewed all the studies they include and found three that reported patient-level data indicating fibrosis progression in liver biopsies[11–13]. We derived the transition probabilities for ARLD progression by fitting a multi-state Markov model to these data. To standardise the histological findings across studies for our model, we mapped the reported fibrosis stages into our three model states, as follows:

- Nakano *et al.* (1982)
  - F0 / F1 : Steatosis
  - F2 / F3 : Steatosis with perivenular fibrosis and fibrosis
  - F4 *:* Incomplete cirrhosis and cirrhosis
- Worner & Lieber (1985)
  - F0 / F1 : Steatosis
  - F2 / F3 : Steatosis with perivenular fibrosis and septal fibrosis
  - F4 *:* Incomplete cirrhosis and cirrhosis
- Marbet *et al.* (1987)
  - F0 / F1 : Fibrosis 0–+
  - F2 / F3 : Fibrosis ++*,* fibrosis +++*, and* early nodular transformation (locally)
  - F4 : Completed nodular transformation (locally)*,* Diffuse early nodular transformation*,* Diffuse early localised established cirrhosis*, and* Diffuse established cirrhosis

We then merged the data into one dataset, which we used to fit the model.

These data represent cross-sectional "snapshots" of patients at various fibrosis stages. We used the {msm} package in R to fit a continuous-time Markov model, assuming homogeneous transition rates between fibrosis states over time.

We modelled disease progression through our three clinically defined states. Given the nature of the data, we specified an initial transition intensity matrix (*Q* matrix) to reflect plausible transitions between states. The *Q* matrix allowed for progression from state 1 (F0/F1) to state 2 (F2/F3), bidirectional transitions between states 1 and 2 to capture potential regression and progression, and progression from state 2 to the absorbing state 3 (F4, cirrhosis), with no transitions from state 3.

We estimated the transition intensities using maximum likelihood estimation and calculated bootstrapped confidence intervals to account for parameter uncertainty. From these intensities, we derived the mean transition probability matrices over relevant time intervals to inform our Markov model. We incorporate 10,000 bootstrapped transition probability matrices directly in the decision model, sampling a randomly selected realisation in each iteration of probabilistic analyses.

This approach allowed us to incorporate empirically derived ARLD natural history progression rates into the health economic model.

3.2. Effectiveness of lifestyle intervention for people with MASLD

Our estimate of the effectiveness of lifestyle intervention for people diagnosed with MASLD comes from Scragg *et al.* (2020), a UK before–after study assessing impact of a very-low-calorie diet on weight-loss and liver health[14]. In our base case, we use effects reported before and after the 8-week intervention. The authors also report findings 9 months after the intervention started (although data were not available for a larger proportion of participants); we explore the effect of using this effect instead in a scenario analysis.

The authors report participants (n=30) had mean LSM of 13.0 (SD 6.6) kPa before intervention, falling to 8.0 (SD 2.9) kPa afterwards. Because 3 people dropped out of the intervention, we adjusted the reported results, assuming that the dropouts would gain no benefit (remain at baseline mean level), resulting in a revised follow-up LSM of 8.5 (SD 3.4) kPa.

To translate these continuous data into the dichotomous effect we need for our model, we use a transformation first derived by Hasselblad and Hedges (1995)[27] and subsequently popularised by Chinn (2000)[28]. First, we calculate a standardised mean difference for the before–after comparison: −0.854 (SE 0.270). Then, on the assumption that the continuous

data have an approximately logistic distribution, we can calculate a log-odds ratio using the formula

$$\ln(OR) = SMD\frac{\pi}{\sqrt{3}}$$

This gives us −1.550 (95%CI: −2.510 to −0.589) which, on the natural scale, is an odds ratio of 0.212 (95%CI: 0.081 to 0.555). This implies that people who receive the intervention have 0.2-times the odds of falling above any given LSM cutoff than they did at baseline. We use this odds ratio to adjust transition probabilities between fibrosis states in the model (weighted according to the proportion of people that we assume would accept the intervention – 28.0%, derived from Taylor *et al.*, 2016[15]).

In the 9-month results, LSM fell further than at 8 weeks among participants for whom data were available (n=20), to 6.9 (SD 2.0) kPa; however, when we perform the same conservative adjustment for missing data detailed above, we get a revised follow-up LSM of 8.9 (SD 4.1) kPa. The odds ratio we derive from this using the Hasselblad–Hedges transformation is 0.262 (95%CI: 0.101 to 0.678).

3.3. Effectiveness of surveillance for hepatocellular carcinoma

We use evidence from Haq *et al.*[20] to estimate the impact of HCC surveillance on survival. First, we calculated that people who develop HCC under (fully or partially adherent) surveillance have a 0.493 probability of having an early-stage tumour (stage 0/A according to the Barcelona Clinic Liver Cancer [BCLC] schema). Then, we estimate the extent to which the odds of presenting at the same stage are lower in people who did not receive surveillance (odds ratio = 0.22 [95%CI 0.16 to 0.31]). Combining these two datapoints gives us a probability of 0.177 that people develop HCC at stage 0/A without surveillance. Next, we extracted data from the Kaplan–Meier curves Haq *et al.* provide showing survival according to BCLC stage of HCC at diagnosis, using Guyot *et al.*'s algorithm[29]. We pooled data for BCLC B and C, as these are handled together in our model. Finally, we fitted an exponential survival model to the pseudo-patient-level data we had extracted. *Table e7* shows the fitted coefficients, and provides a variance–covariance matrix, which we used to characterise uncertainty in probabilistic analyses (multivariate normal sampling via Cholesky decomposition).

Rogers G, *et al. BMJPH* 2026; 4:e003480. doi: 10.1136/bmjph-2025-003480

Table e7 Exponential survival model fitted to data extracted from Haq *et al.*[20]

Term	Estimate (95% confidence interval)	Variance–covariance matrix		
		Ln(rate)	Ln($HR_{B/C\text{-}v\text{-}0/A}$)	Ln($HR_{D\text{-}v\text{-}0/A}$)
Ln(rate)	−1.798	0.00714	−0.00714	−0.00714
Ln($HR_{B/C\text{-}v\text{-}0/A}$)	1.356	−0.00714	0.00916	0.00714
Ln($HR_{D\text{-}v\text{-}0/A}$)	2.441	−0.00714	0.00714	0.01249
Exponentiated				
Rate (BCLC 0/A)	0.166 (0.140 to 0.196)			
Hazard ratios				
BCLC 0/A	1			
BCLC B/C	3.88 (3.22 to 4.68)			
BCLC D	11.49 (9.23 to 14.30)			

We use an exponential function as it provides a visually adequate fit to the data and, because members of the simulated cohort develop HCC throughout the modelled period, it would not be possible to use a time-varying hazard without substantially increasing model complexity (e.g. with numerous tunnel states or multidimensional transition probability matrices). *Figure e1* illustrates the underlying data and the fitted exponential functions.

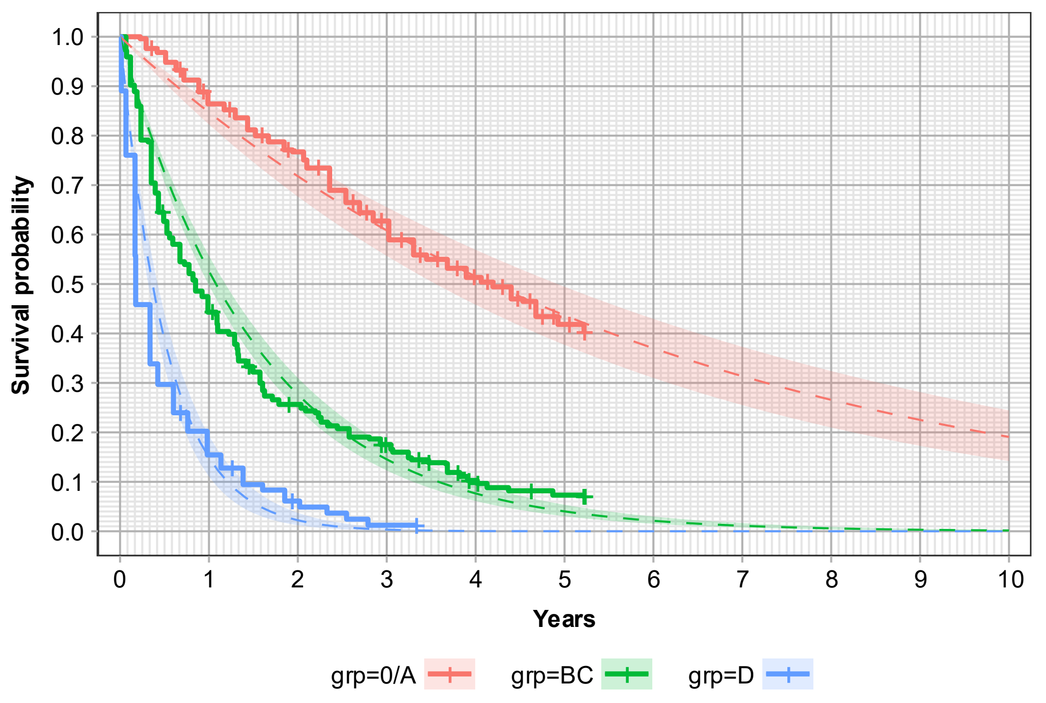

Solid, stepped lines show empirical survival data (reconstructed from original publication). Dashed, smooth lines show fitted exponential curves. Shaded bands show 95% confidence intervals for fitted curves.

Figure e1 Survival data reconstructed from Haq *et al.*[20] with overlaid fitted exponential curves

eAppendix 4 Derivation of model parameters – costs

4.1. Upfront costs

4.1.1. Reactive referral

Unit costs for the reactive referral pathway are derived from PSSRU 2023/24 and NHS Cost Collection 2023/24[22,23].

In the reactive referral pathway, we account for primary and secondary care costs. The costs incurred in primary care comprise two GP appointments including a full liver-screen aetiology blood-test and a directly-accessed ultrasound. The unit cost of a standard 10-minute GP appointment is sourced from PSSRU 2023/24[23]. For the cost of the ultrasound, we calculate a weighted average of ultrasound scan costs with a procedural duration above and below 20 minutes. Ultrasound and blood test unit costs are obtained from NHS Cost Collection 2023/24[22]. In total, this amounts to **£181.47**.

Following the results of the diagnostic investigations, patients are initially assessed through a 5-minute consultant-led qualitative triage in secondary care. The hourly cost of a consultant is derived from PSSRU 2023/24[23]. Those stratified to be at risk of significant CLD attend a one-stop outpatient hepatology appointment, including transient elastography. We derive the cost of a first, consultant-led, in-person outpatient appointment from NHS Cost Collection 2023/24[22]. For elastography, we account for equipment costs (sourced from NICE DG48) and 10 minutes of nurse-time (average of band 6 and band 7 pay from PSSRU 2023/24[23])

This amounts to a total of **£295.74**. The whole reactive referral pathway including primary and secondary care costs comes to £477.22.

Rogers G, *et al. BMJPH* 2026; 4:e003480. doi: 10.1136/bmjph-2025-003480

Table e8 Costs associated with reactive referral

Item	Cost	Notes	Source
Primary care			
GP appointments	£90.00	Assume average of 2 appointments before referral	PSSRU 2023/24[23]
Liver screen blood tests	£20.49	Sum of HRG codes: PATH04 (Clinical biochemistry), PATH05 (Haematology), PATH06 (Immunology), PATH07 (Microbiology)	National cost collection 2023/24[22]
Ultrasound	£70.98	Directly accessed ultrasound. Weighted average of RD40Z (duration <20min, without contrast) and RD42Z (duration ≥20min, without contrast)	National cost collection 2023/24[22]
Primary care total	£181.47		
Secondary care			
Consultant qualitative triage	£8.58	Assume 5min per case	PSSRU 2023/24[23]
Transient elastography	£38.08	Assumes a 7-year lifetime of a single device, delivering an average of 610 scans per year (average of data from 4 NHS hospitals, including Wythenshawe, Greater Manchester). Accounts for purchase price, 1 additional probe, licence fees for software, service contract, and training costs (all provided to NICE by Echosens). No depreciation. (£28.11; inflated from 2019/20 cost-year)	NICE DG48
Transient elastography nurse time	£11.25	Assume 10min per case	PSSRU 2023/24[23]
Hepatology outpatient appointment	£242.82	WF01B: Consultant-led, Non-Admitted Face-to-Face Attendance, First	National cost collection 2023/24[22]
Secondary care total	£295.74		
**Whole reactive referral pathway**	**£477.22**		

4.1.2. Proactive case-finding

The costs in the proactive referral pathway account for the community-based case-finding efforts which entail using routine primary-care data to identify patients with risk factors for CLD, inviting them to undergo health checks and referring them to the ID-LIVER team.

4.1.2.1. Finding people at risk

Current ADA grant (base-case approach)

In the base case of the model, we calculate the cost for proactive case-finding on the basis of our current experience in a successor project to ID-LIVER, the Advanced Diagnostics Accelerator (ADA). Instead of using region-wide electronic searches, as in ID-LIVER, we now work with local GP practices to identify people on their records with one or more risk factor for CLD. Practices are paid a maximum of £5,000 for their participation in ADA (including running searches, sending out invitations by SMS and/or letter, and referring patients who opt in to the CLAC team). To calculate cost per person identified, we simply divide this maximum grant by the average number of people who opt in to attend CLACs per

Rogers G, et al. BMJPH 2026; 4:e003480. doi: 10.1136/bmjph-2025-003480

Supplemental material BMJ Publishing Group Limited (BMJ) disclaims all liability and responsibility arising from any reliance placed on this supplemental material which has been supplied by the author(s) BMJPH

practice. Currently, this figure stands at 67, so we estimate the cost per person identified to be **£74.63**.

We exclude one GP practice (Northenden Group Practice) from this calculation, as ID-LIVER investigators worked closely with practice staff while piloting the approach. This resulted in a high level of buy-in that we accept is unrealistic in routine circumstances. If we were to include Northenden Group Practice in our estimates, the cost per person identified would fall to £47.47.

Micro-costing

As part of the ID-LIVER project, we worked directly with GP practices, collecting cost data on the proactive case-finding process. A local GP practice, Northenden Group Practice, provided observed cost data on the admin time to searches, update the website, work on patient invitations, post letters, and pass on details of people who chose to opt in to the CLAC team. This amounts to a total of £1,092. The practice invited 1,000 people, of whom 181 opted in to attend the CLAC. We also account for the cost of GP time to run patient searches for which the reported duration was 30 minutes. To calculate the cost per person identified, we divide the total reported spend by the patient yield at the practice, arriving at a cost of **£6.44**.

Table e9 Costs associated with finding people at risk of liver disease

Item	Value	Notes	Source
GP time to run searches (mins)	30	Actioning searches	Personal communication from Northenden Group Practice
GP practice admin	£1,092	Patient invitations, letter postage, website updates.	
GP time per hour	£178		PSSRU 2023/24[23]
Total cost	£1,181		
Patients seen	182		ID-LIVER
**Cost per person identified**	**£6.44**		

ID-LIVER research costs (scenario analysis)

It is a matter of public record that NorthWest EHealth Limited, the project partner who undertook the electronic searches for ID-LIVER, received £50,982 for their contribution to the research project. Given the final yield of 296 people attending CLACs during the funded project, this amounts to a cost per person identified of **£172.24**. We explore the impact of using this cost in a scenario analysis, as it factually represents the expenditure in the project. However, we have subsequently demonstrated that, by working directly with GP practices, we

Rogers G, et al. BMJPH 2026; 4:e003480. doi: 10.1136/bmjph-2025-003480

can achieve the same thing at substantially lower cost (see above), so we take the view that the research cost is not representative of expected expenditure in practice.

4.1.2.2. Community liver assessment clinic

Micro-costing

In the micro-costing exercise for the community liver assessment clinics (CLACs), we calculate the costs of assessing patients referred to a CLAC following identification. These costs account for the clinic location which either entails the use of local healthcare facilities or a mobile screening van. In ID-LIVER, a mobile screening van was used at 4 clinic sites, over 28 days in total across all sites, during which 238 patients were seen. The costs include a one-off set-up and admin fee per site, and a daily van rate. Alternatively, the costing for CLACs using local GP facilities account for 9 days of room hire, and 2 admin staff working 7.5 hours daily. These costs are based on observed data from an ID-LIVER GP site where 182 patients attended a CLAC.

For both clinic locations, we account for staff time for a hepatologist (registrar) and hepatology nurse based on a 30-minute appointment duration during which patients receive a blood test and undergo transient elastography. Staff-time unit-costs for a hepatology registrar and nurse (average of band 6 and band 7 pay) come from PSSRU 2023/24[23]. We source the costs of a liver screen blood test from NHS Cost Collection 2023/24[22] and transient elastography equipment from NICE DG48. The appointment duration, room hire, admin time, and mobile van screening costs represent real-world estimates based on observations from the ID-LIVER project. Considering the patient yield for the mobile screening vans and local GP CLACs, the estimated cost per person amounts to **£153.33** and **£146.60** respectively. *Table e10*Table provides details.

Table e10 Costs associated with community liver assessment clinics

Item	Cost	Notes	Source
Staff time and tests per person seen			
Hepatology registrar time (hours)	0.5		Authors' assumptions, based on experience in ID-LIVER
Nurse time (hours)	0.5	In theory, nurses can deliver elastography in less time than this (we estimate a usual duration of 10 minutes). However, we find that, in space-restricted settings, it is not possible to run multiple activities concurrently while maintaining patient confidentiality. Therefore, we make the assumption that both the hepatologist and the nurse are effectively committed to a single patient for the duration of each 30-minute appointment.	

Hepatology registrar per hour	£79.00		PSSRU 2023/24[23]
Nurse per hour	£67.50	Average of band 6 and band 7	
Transient elastography equipment costs			
Pay-per-scan business model	£68.26	Provided to NICE by Echosens (£58; inflated from 2019/20 cost-year)	NICE DG48
Outright purchase	£33.08	Assumes a 7-year lifetime of a single device, delivering an average of 610 scans per year (average of data from 4 NHS hospitals, including Wythenshawe, Greater Manchester). Accounts for purchase price, 1 additional probe, licence fees for software, service contract, and training costs (all provided to NICE by Echosens). No depreciation. (£28.11; inflated from 2019/20 cost-year)	
Liver screen blood test	£20.49	Sum of HRG codes: PATH04 (Clinical biochemistry), PATH05 (Haematology), PATH06 (Immunology), PATH07 (Microbiology)	National cost collection 2023/24[22]
Total staff time and tests			
Assuming pay-per-scan elastography	£162.00		
Assuming outright purchase	£126.82		
Consultation facilities			
Option 1: mobile screening van			
Set-up fee per site	£256	Driver time, set-up/close-down, training	Personal communication, Manchester University NHS Foundation Trust Research & Innovation
Admin fee per site	£250	Admin set-up process	
Daily van rate	£153	Running costs, petrol, equipment usage	
Number of clinic sites	4		ID-LIVER
Total number of days	28		
Total van costs	£6,308		
Total number of patients seen	238		
Cost per person (screening van)	£26.50		
Option 2: room hire			
Daily room hire	£175	Paid daily	Personal communication from Northenden Group Practice
Reception / admin staff per day	£225	2 admin staff at £15 per hour for 7.5 hours	
Number of days	9		ID-LIVER
Total room hire / admin staff cost	£3,600		
Total number of patients seen	181		
Cost per person (room-hire)	£19.89		
**Total cost per person**			
**Base case**	**£153.33**	Assumes outright purchase of transient elastography equipment and mobile screening van consultation costs	
Most expensive scenario	£188.51	Pay-per-scan transient elastography equipment and mobile screening van consultation costs	

National HRGs

As a simple alternative to our micro-costing, we also explored using national HRGs for a hepatology appointment and transient elastography costs (i.e. exactly the same as specified for the secondary care component of the reactive referral pathway excluding the consultant-led qualitative triage). This amounts to £287.16 per person seen.

4.2. Ongoing fibrosis-stage-specific hepatology costs

Patients diagnosed with F2/3 fibrosis are referred to secondary care. The costs for this health state are estimated to be a total of **£309.96** per year. This accounts for a single hepatology outpatient appointment per year during which patients receive a full liver screen blood test

and transient elastography. The costs of the appointment, blood test, and transient elastography are derived from NHS Cost Collection 2023/24[22].

We derive costs for cirrhosis from NICE guideline NG50. We inflate the 6-monthly compensated and decompensated cirrhosis health-state costs for MASLD and ARLD to 2023/24 prices and double them to arrive at the equivalent cost per yearly cycle. For compensated cirrhosis, these costs comprise of a hepatologist appointment and a combination of tests including a full blood count, international normalized ratio and liver blood test. This amounts to **£475.71** for both ARLD and MASLD patients. Costs for decompensated cirrhosis account for three hepatologist appointments, the same combination of tests and include complication costs. In total, this amounts to **£24,169.21** for ARLD patients and **£16,612.81** for MASLD patients. As we only account for liver-related resource use, people in the F0/F1 health states and those who are undiagnosed incur no costs.

Table e11 Costs of ongoing hepatology care

Health state	Cost	Notes	Source
MASLD			
F0/1	£0.00		
F2/3	£309.96	1 hepatologist outpatient appointment, full liver screen blood test, and transient elastography.	NHS Cost Collection 2023/24[22]
Compensated cirrhosis	£475.71	1 hepatologist appointment, full blood count, international normalized ratio test, and liver blood test. Inflated from 2013/14 to 2023/24 prices and doubled (NICE model has 6-month cycle where ours in 12-months).<br>Surveillance for HCC added separately for a proportion of people; see below.	NICE guideline NG50
Decompensated cirrhosis	£16,612.81	3 hepatologist appointments, full blood count test, international normalized ratio test, liver blood test, and complication costs. Inflated from 2013/14 to 2023/24 prices and doubled (NICE model has 6-month cycle where ours in 12-months).	NICE guideline NG50
ARLD			
F0/1	£0.00		
F2/3	£309.96	1 hepatologist outpatient appointment, full liver screen blood test, and transient elastography.	NHS Cost Collection 2023/24[22]
Compensated cirrhosis	£475.71	1 hepatologist appointment, a full blood count test, international normalized ratio test, and liver blood test. Inflated from 2013/14 to 2023/24 prices and doubled (NICE model has 6-month cycle where ours in 12-months).	NICE guideline NG50
Decompensated cirrhosis	£24,169.21	3 hepatologist appointments, a full blood count test, international normalized ratio test, liver blood test, and 50% increased complication costs. Inflated from 2013/14 to 2023/24 prices and doubled (NICE model has 6-month cycle where ours in 12-months).	NICE guideline NG50

ARLD = alcohol-related liver disease; F0/1/2/3/4 = METAVIR fibrosis stage; MASLD = metabolic-dysfunction-associated steatotic liver disease

4.2.1. Surveillance for HCC

For the patients undergoing HCC surveillance, we account for the cost of each visit. Visits comprise of a clinical biochemistry blood test and a directly accessed ultrasound. These unit

Rogers G, et al. BMJPH 2026; 4:e003480. doi: 10.1136/bmjph-2025-003480

costs are both sourced from NHS Cost Collection 23/24[22]. The visit frequency is determined by the level of adherence, with two visits yearly for adherent patients and one for non-adherent patients. The probability of adherence is estimated to be 77% as derived from Haq et al.[20] which looked at adherence to HCC surveillance and its impact on survival in a UK mixed aetiology cirrhosis population. The estimated total cost per year is £121.14.

Table e12 Costs associated with surveillance for HCC

Item	Cost	Notes	Source
Clinical biochemistry blood test	£1.53		NHS Cost Collection 2023/24[22]
Outpatient ultrasound scan	£67.05	Weighted average of ultrasound procedure costs lasting over and below 20 minutes.	NHS Cost Collection 2023/24[22]
Cost per visit	£68.58		
Total cost per year	£121.14	Calculated based on a 77% probability of adherence (Haq et al., 2021), with adherent patients attending two appointments and non-adherent patients attending one.	

4.3. Lifestyle intervention costs

Patients identified to have MASLD with significant or more severe fibrosis are referred to a lifestyle intervention in form of a dietary intervention. Depending on the uptake level, the incurred costs account for the very low-calorie diet (VLCD) offered to patients. We calculate a mean price per serving using information on the prices and servings of the recommended meal replacement products. Unit costs for these products are obtained from Optifast[30]. Using assumptions about the servings per week derived from Scragg et al.[14], we subsequently estimate the total price of the diet. Additionally, we account for staff time for a band 7 Dietitian per NICE guideline CG189, assuming patients also attend 12 dietitian appointments each for the duration of 30 minutes. Staff costs are obtained from PSSRU 2023/24[23], and assumptions about the number of and duration of the appointments from NICE guideline CG189. The total intervention cost amounts to £1,207.69, which gives an average cost of £416.19 per diagnosed person based on our estimate that 28.0% of people will choose to accept the intervention[31].

For ARLD patients, we account for staff time for a band 7 clinical psychologist, assuming patients who accept treatment undergo a 12-appointment behavioural intervention, each for the duration of an hour. Staff costs are also obtained from PSSRU 2023/24[23] with assumptions about appointment frequency and duration sourced from NICE CG115. This adds up to a total intervention cost of £861.54 resulting in an average cost of £252.16 based on a 29.3% uptake level[19]. In a scenario analysis, we also include the costs of pharmacological intervention; this raises the cost per person to £369.74.

Rogers G, et al. BMJPH 2026; 4:e003480. doi: 10.1136/bmjph-2025-003480

Table e13 Costs associated with lifestyle interventions

Item	Cost	Notes	Source
MASLD (very low-calorie diet)			
Meal replacement products (per serving)	£2.69	Calculated by dividing the price per pack of each Optifast meal replacement product by the servings per pack	Optifast website
Total price	£791.49	294 servings over 12 weeks	Scragg *et al.* (2021)
Dietitian cost	£416.19	12 × 30-minute appointments with band 7 dietitian	PSSRU 2023/24[23]<br>NICE CG189
Total intervention cost	£1,207.69		
Mean intervention cost per diagnosed person	£338.31	Assumes 28.0% uptake	[15]
ARLD (behavioural intervention)			
Total intervention cost	£861.54	12 × 1-hour appointments with band 7 clinical psychologist	PSSRU 2023/24[23]<br>NICE CG115
Mean intervention cost per diagnosed person	£252.16	Assumes 29.3% uptake	[19]
ARLD (behavioural and pharmacological intervention – scenario analysis)			
Course of naltrexone	£434.10	50mg × 28 @ £72.35 × 6	NICE CG115<br>NHS Drug Tariff<br>March 2024
Course of acamprosate	£132.12	333mg × 168 @ £22.02 × 6	NICE CG115<br>NHS Drug Tariff<br>March 2024
Ratio of naltrexone:acamprosate	0.893:0.107		NHS Prescription Cost Analysis England 2024/25
Total cost	£1,263.29	Behavioural plus pharmacological	
Mean intervention cost per diagnosed person	£369.74	Assumes 29.3% uptake	[19]

ARLD = alcohol-related liver disease; MASLD = metabolic-dysfunction-associated steatotic liver disease

Rogers G, *et al. BMJPH* 2026; 4:e003480. doi: 10.1136/bmjph-2025-003480

4.4. HCC treatment

Table e14 Costs associated with treatment for HCC

Item	Costs	Notes	Source
Compensated cirrhosis			
Ablation and curative	£13,759	Obtained 2-year mean cost estimates from authors of Cullen et al. (2023), inflated these values from 2018/19 to 2023/24 prices and divided by two to derive the per-cycle treatment cost. In probabilistic analysis, we assume SE is 20% of mean, in absence of empirical estimates of uncertainty.	Cullen et al. (2023)[21]
Ablation and palliative	£18,405		
Initial palliative and curative	£27,851		
Liver resection and other	£11,637		
Liver transplant and other	£37,910		
Cytotoxic chemotherapy and other	£12,739		
Other palliative and other	£9,794		
TACE and other	£15,230		
No active treatment	£4,802		
Decompensated cirrhosis			
Initial palliative and curative	£35,779		
Other palliative and other	£11,686		
No active treatment	£4,832		
Systemic therapy			
Progression free	£28,498.12	Based on NICE appraisals, unit costs updated or values inflated	NICE TA474
Post-progression	£37,562.72		NICE TA551
Proportion of people receiving each treatment			
TNM stage I (~= BCLC 0/A)		Probabilistic parameters: Dirichlet (α_1=229, α_2=25, α_3=157, α_4=164, α_5=208, α_6=82)	Driver (2022)[32]
Best supportive care	0.2647		
Sorafenib	0.0289		
TACE	0.1815		
Ablation	0.1896		
Resection	0.2405		
Transplantation	0.0948		
TNM stage II-IV (~= BCLC B/C)		Probabilistic parameters: Dirichlet (α_1=2,523, α_2=385, α_3=487, α_4=132, α_5=323, α_6=103)	
Best supportive care	0.6382		
Sorafenib	0.0974		
TACE	0.1232		
Ablation	0.0334		
Resection	0.0817		
Transplantation	0.0261		
BCLC D			
Best supportive care	1.0000	Assumption	
Costs per person per year			
BCLC 0/A			
Year 1	£15,018.93	Weighted averages of the above	
Year 2	£15,128.52		
Year 3 onwards	£4,801.62	Assume as for no active treatment	
BCLC B/C			
Year 1	£12,639.41	Weighted averages of the above	
Year 2	£13,008.73		
Year 3 onwards	£4,801.62	Assume as for no active treatment	
BCLC D			
Year 1 onwards	£21,739.80	BSC costs plus post-decompensation costs (see *Table e11*Table)	

BCLC = Barcelona Clinic Liver Cancer stage; BSC = best supportive care; TACE = transarterial chemoembolization; TNM = Tumour–Node–Metastasis classification of malignant tumours

Rogers G, *et al. BMJPH* 2026; 4:e003480. doi: 10.1136/bmjph-2025-003480

eAppendix 5 Derivation of model parameters – health-related quality of life

5.1. No fibrosis or compensated fibrosis

We use EQ-5D-5L measurements from the ID-LIVER cohort, crosswalked to EQ-5D-3L values[33], to estimate health-related quality of life (HRQoL) in people with compensated CLD; *Table e5* gives results. As well as dividing according to aetiology of liver disease (ARLD -v- MASLD), we split HRQoL values derived from the ID-LIVER cohort according to level of fibrosis (F0/F1 -v- F2/F3/F4). We explored introducing an additional distinction between F2/F3 disease and compensated cirrhosis (F4), in line with the states in our natural history model. However, there was no evidence of difference in EQ-5D-3L utility between people with F2/F3 disease and those with F4; this is consistent with the observation that compensated CLD is largely asymptomatic.

We compared observed EQ-5D-3L utility with expected utility for the general population as reported by Hernández Alava *et al.*[24], age- and sex-matched for each category. Dividing the former by the latter provides a multiplier that we apply in the model to expected utility for the cohort as it ages, which enables us to account for decreasing HRQoL with age. In probabilistic analyses, we incorporate uncertainty in the estimates of general population utility by sampling coefficients for Hernández Alava *et al.*'s model from a multivariate normal distribution, using the variance–covariance matrix the authors provide. We then recalculate the relevant multipliers for each iteration of the probabilistic model.

In our base case, we assume that people retain the HRQoL associated with their baseline fibrosis-state, even if their fibrosis worsens or improves, until they experience decompensation or HCC diagnosis. This is because compensated CLD is largely asymptomatic, and we did not want to assume a causal relationship between liver pathology and HRQoL, when the association between the two is likely mediated by other factors (e.g. comorbidities; extent of obesity / harmful alcohol use). However, the lifestyle interventions that we simulate may also have an impact on these factors, so we explore the impact of allowing HRQoL to vary with fibrosis-state in a scenario analysis.

5.2. Decompensated cirrhosis

Table e15 shows how we estimate health-related quality of life (utility) for people with decompensated cirrhosis. By pooling the ratio of means in 10 studies reporting EQ-5D

Rogers G, et al. BMJPH 2026; 4:e003480. doi: 10.1136/bmjph-2025-003480

utilities for populations with compensated and decompensated cirrhosis, we estimate a multiplier, which we then apply to the relevant age- and sex-adjusted utility for people with F2/F3/F4 fibrosis (see main text).

Table e15 Health-related quality of life associated with decompensated cirrhosis compared with compensated cirrhosis

Study	Country	Population	Compensated		Decompensated		Multiplier (ratio of means)
			N	Utility (95%CI)	N	Utility (95%CI)	
Bjornsson et al., 2009[34]	Sweden	HCV	76	0.749 (0.700 to 0.795)	53	0.656 (0.583 to 0.726)	0.876 (0.772 to 0.994)
Chong et al., 2003[35]	Canada	HCV	24	0.750 (0.660 to 0.830)	9	0.660 (0.448 to 0.843)	0.880 (0.637 to 1.216)
Cortesi et al., 2020[36]	Italy	CLD	574	0.891 (0.881 to 0.901)	523	0.859 (0.847 to 0.871)	0.964 (0.947 to 0.981)
Pol et al., 2015[37]	France, Germany, UK	HCV	101	0.670 (0.610 to 0.727)	25	0.510 (0.373 to 0.646)	0.761 (0.574 to 1.010)
Samp et al., 2015[38]	France	HCV	18	0.622 (0.532 to 0.708)	11	0.405 (0.288 to 0.528)	0.651 (0.468 to 0.905)
Sugimori et al., 2022[39]	Japan	HBV	141	0.845 (0.818 to 0.870)	35	0.722 (0.646 to 0.792)	0.854 (0.769 to 0.950)
		HCV	260	0.737 (0.713 to 0.760)	96	0.671 (0.625 to 0.715)	0.910 (0.845 to 0.980)
Vargas et al., 2015[40]	Chile	HCV	9	0.682 (0.118 to 0.996)	2	0.536 (0.025 to 0.988)	0.786 (0.209 to 2.954)
Vellopoulou et al., 2014[41]	Netherland	HCV	23	0.730 (0.649 to 0.804)	4	0.500 (0.302 to 0.698)	0.685 (0.453 to 1.036)
Woo et al., 2012[42]	Canada	HBV	79	0.880 (0.843 to 0.913)	7	0.730 (0.379 to 0.962)	0.830 (0.545 to 1.262)
Wright et al., 2006[43]	UK	HCV	40	0.550 (0.444 to 0.654)	64	0.450 (0.365 to 0.537)	0.818 (0.624 to 1.073)
**Pooled (random-effects meta-analysis)** χ^2=20.87; df = 10; p=0.022; τ^2=0.004; I^2=52.1%							**0.879 (0.824 to 0.939)**

CLD = chronic liver disease; HBV = hepatitis B virus; HCV = hepatitis C virus

5.3. Hepatocellular carcinoma

We use evidence from Verma *et al.*[25] to account for the impact of early- and late-stage HCC diagnosis on HRQoL. They provide SF-36 measurements before and after diagnosis, distinguishing between people who developed stage I-II and stage III-IV tumours (according to the American Joint Committee on Cancer [AJCC] staging system – analogous to the T-stage of TNM). We map domain-level SF-36 data onto EQ-5D-3L values using model 6 from Ara and Brazier's algorithm[44].

Before HCC diagnosis, participants who went on to develop HCC had EQ-5D-3L utility of 0.545. Those who developed stage I/II HCC in the subsequent 2 years had EQ-5D-3L utility of 0.535 (equivalent to a utility multiplier of 0.982, compared with baseline); people with stage III/IV HCC had EQ-5D-3L utility of 0.480 (utility multiplier = 0.880, compared with baseline).

In the model, we apply these multipliers to the utility values for F2/F3/F4 fibrosis (which, in turn, we derive using a multiplier for fibrosis compared with expected utility in age- and sex-matched population; see main text). We assume the value for stage I/II HCC is relevant for people with BCLC 0/A disease, and the value for stage III/IV HCC applies to people with BCLC B/C/D tumours. For people with underlying decompensated cirrhosis (i.e. BCLC stage D HCC), we also apply the multiplier described in *5.1*, above.

In probabilistic analyses, we account for uncertainty in these quantities by sampling values for the domain-level SF-36 scores and the associated SF-36–EQ-5D-3L mapping coefficients from independent normal distributions. Ideally, we would account for correlations in both steps in multivariate sampling; however, information on covariance is unavailable in either case.

Rogers G, *et al. BMJPH* 2026; 4:e003480. doi: 10.1136/bmjph-2025-003480

eAppendix 6 State occupancy graphs for each starting state

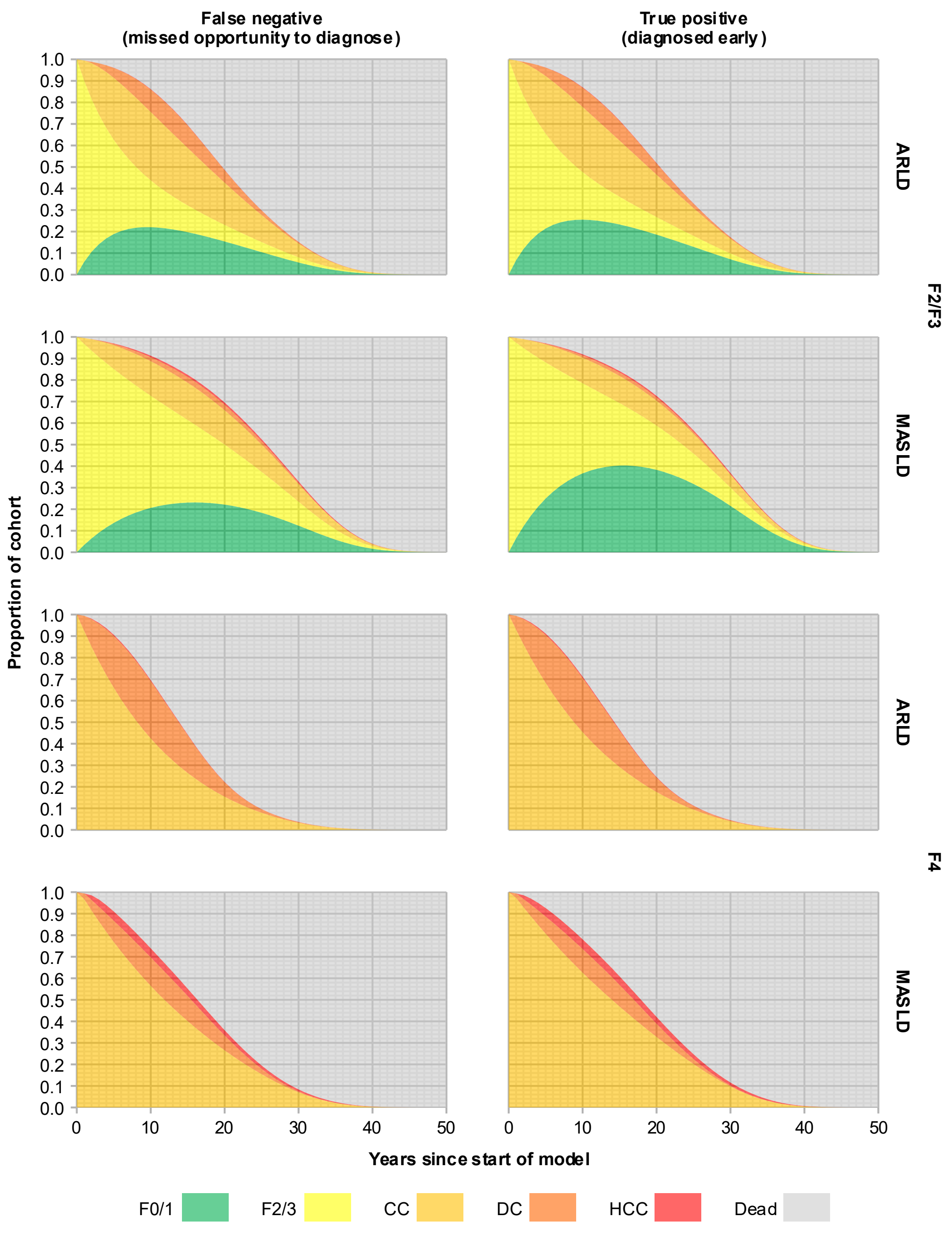

ARLD = alcohol-related liver disease; CC = compensated cirrhosis; DC = decompensated cirrhosis; F0/1/2/3/4 = METAVIR fibrosis stage; HCC = hepatocellular carcinoma; MASLD = metabolic-dysfunction-associated steatotic liver disease

See Table e1 for starting ages of each cohort.

Figure e2 State occupancy over time for people with significant fibrosis, according to aetiology (ARLD -v- MASLD) and diagnosis status (FN -v- TP)

eAppendix 7 Cost effectiveness of hypothetical strategies for identifying people with liver disease

Table e16 Expected lifetime life-years, quality-adjusted life-years, and hepatology costs for people with different levels of fibrosis, according to whether their disease is detected

	Absolute			Incremental, TP -v- FN			Maximum justifiable cost per TP identified[b]
	LYs	QALYs[a]	Costs[a]	LYs	QALYs[a]	Costs[a]	
**ARLD**							
Minimal fibrosis (F0/F1)							
TN or FP	25.78	11.74	£10,702				
Significant fibrosis (F2/F3)							
FN	20.57	8.87	£26,597				
TP	21.21	9.06	£27,490	0.64	0.20	£892	£3,009
Compensated cirrhosis (F4)							
FN	15.04	6.93	£70,391				
TP	15.52	7.10	£71,708	0.47	0.17	£1,316	£2,182
F2 or worse							
FN	18.43	8.12	£43,558				
TP	19.01	8.30	£44,614	0.58	0.19	£1,056	£2,689
**MASLD**							
Minimal fibrosis (F0/F1)							
TN or FP	29.17	13.01	£1,702				
Significant fibrosis (F2/F3)							
FN	25.20	10.12	£6,123				
TP	26.05	10.35	£7,729	0.85	0.23	£1,607	£2,943
Compensated cirrhosis (F4)							
FN	17.34	7.55	£31,860				
TP	18.71	8.03	£34,293	1.37	0.47	£2,434	£6,972
F2 or worse							
FN	23.70	9.63	£11,033				
TP	24.65	9.90	£12,797	0.95	0.27	£1,764	£3,712
**Weighted average**							
Minimal fibrosis (F0/F1)							
TN or FP	28.47	12.75	£3,551				
Significant fibrosis (F2/F3)							
FN	23.60	9.69	£13,179				
TP	24.38	9.91	£14,539	0.78	0.22	£1,360	£2,966
Compensated cirrhosis (F4)							
FN	15.99	7.19	£54,556				
TP	16.83	7.48	£56,332	0.84	0.30	£1,775	£4,150
F2 or worse							
FN	21.38	8.96	£25,261				
TP	22.18	9.20	£26,743	0.80	0.24	£1,482	£3,312

[a] Discounted 3.5% per year

[b] When QALYs are valued at £20,000 each. Any case-identification strategy that finds people with the specified level of fibrosis at a cost per true-positive less than this will have positive net benefit (i.e. an ICER better than £20,000/QALY, compared with no case-identification).

ARLD = alcohol-related liver disease; FN = false negative; FP = false positive; LYs = life-years; MASLD = metabolic dysfunction-associated liver disease; QALYs = quality-adjusted life-years; TN = true negative; TP = true positive

*Table e16*Table shows expected lifetime costs and QALY for true-positives and false-negatives in ARLD, MASLD, and mixed populations. There is no population in which detection of disease leads to net cost-savings. This is because, although we reduce downstream healthcare costs (decompensation; HCC) by slowing progression of liver-disease, expenditure on lifestyle intervention and ongoing follow-up slightly outweigh this saving. However, there is substantial health-gain associated with detection (approximately 0.2 QALYs for people with ARLD; approximately 0.3 QALYs for people with MASLD). If we value QALYs at £20,000 each (as per NICE's lower cost-effectiveness threshold), any programme that detects significant liver-disease at a cost of less than £3,300 per case would generate positive net benefit.

Armed with this information, we can estimate the cost-effectiveness of any strategy for which we know sensitivity, specificity and up-front costs (see *Figure e3*Figure).

Rogers G, *et al. BMJPH* 2026; 4:e003480. doi: 10.1136/bmjph-2025-003480

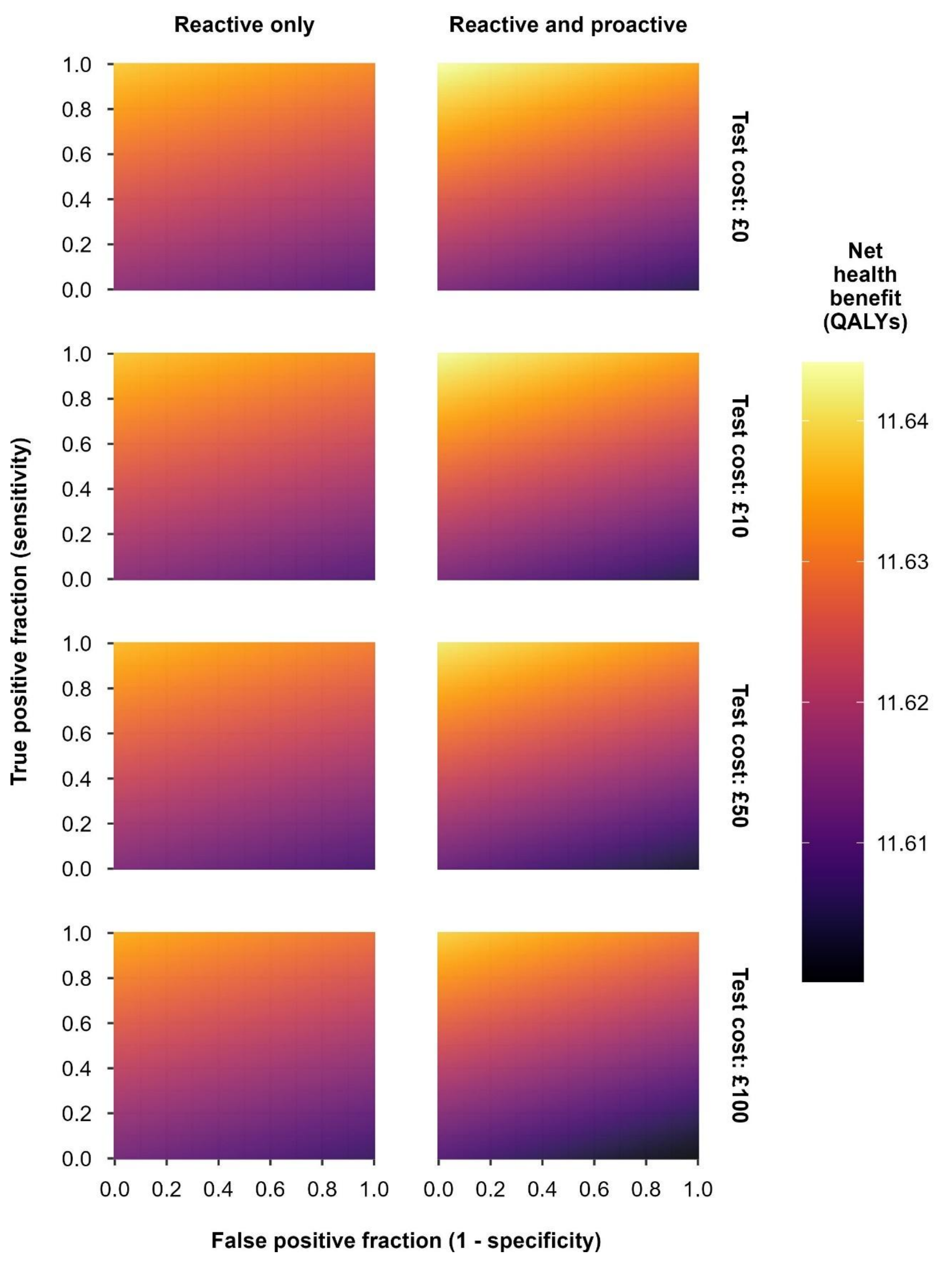

Figure e3 Value for money for risk-stratification strategies with any combination of sensitivity and specificity, at a range of possible test costs

eAppendix 8 Cost-effectiveness results using the previously suggested ID-LIVER-ML threshold of 0.47.

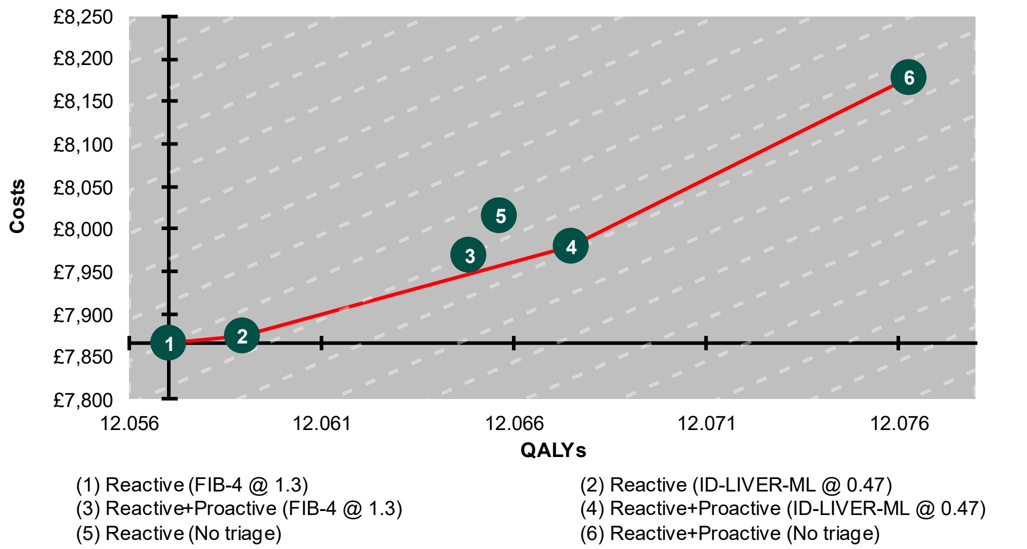

Strategy: Case-finding	Strategy: Risk-stratification (threshold)	Strategy-level sensitivity	Absolute: Costs (£)	Absolute: Effects (QALYs)	Incremental: Costs (£)	Incremental: Effects (QALYs)	Incremental: ICER (£/QALY)	Net health benefit: £20K/ QALY	Net health benefit: £30K/ QALY
Reactive	FIB-4 (1.3)	56.4%	£7,865	12.057				11.664	11.795
Reactive	ID-LIVER-ML (0.47)	61.0%	£7,874	12.059	£8	0.002	£4,250	11.665	11.796
Reactive+Proactive	FIB-4 (1.3)	74.3%	£7,970	12.065	£96	0.006	ext. dom.	11.666	11.799
Reactive+Proactive	ID-LIVER-ML (0.47)	80.8%	£7,980	12.068	£107	0.009	£12,449	11.668	11.802
Reactive	None	75.4%	£8,015	12.066	£35	-0.002	dom.	11.665	11.798
Reactive+Proactive	None	100.0%	£8,179	12.076	£199	0.009	£22,626	11.667	11.804

Dom = dominated; Ext. dom. = extendedly dominated; FIB-4 = Fibrosis-4 index; ID-LIVER-ML = ID-LIVER machine-learning risk-prediction model; QALY = quality-adjusted life-years

Solid red line shows cost-effectiveness frontier. Dashed line in background shows iso-net-benefit gradient equal to £20,000 per QALY. If the line between any two points is shallower than this gradient, the option on the right (generating most QALYs) represents good value; if it is steeper, the option on the left is better value, assuming we value QALYs at £20,000 each.

Sub-table shows fully incremental analysis (i.e. incremental values reflect the comparison between each option and the next-cheapest non-dominated alternative).

All outcomes represent expected values per person within the whole at-risk population. All outcomes discounted at 3.5% per year.

Figure e4 Cost effectiveness of different approaches to case-finding and risk-stratification as assessed in ID-LIVER when the ID-LIVER-ML threshold is set to the suggested value of 0.47

BMJ Publishing Group Limited (BMJ) disclaims all liability and responsibility arising from any reliance placed on this supplemental material which has been supplied by the author(s)

eAppendix 9 Threshold analyses

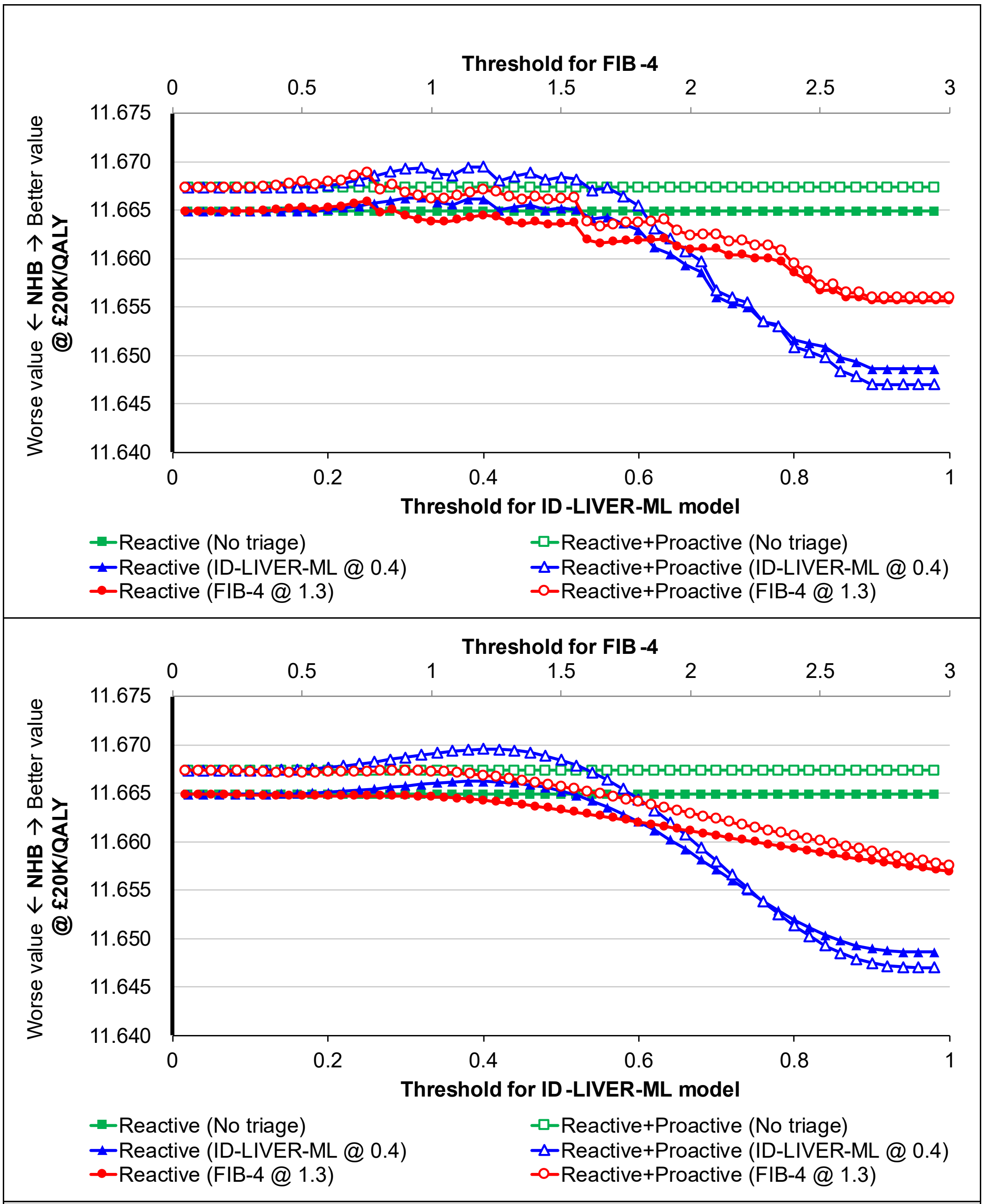

FIB-4 = Fibrosis-4 index; ID-L-ML = ID-LIVER machine-learning risk-prediction model; NHB @ £20K/QALY = net health benefit when we value quality-adjusted life-years at £20,000 each

Upper panel shows results when we use patient-level data to estimate diagnostic accuracy; lower panel shows results using a beta regression fitted to those data

Figure e5 Threshold analysis: relationship between diagnostic threshold (cutoff) for FIB-4 and ID-LIVER-ML model and value for money (net health benefit)

Rogers G, *et al. BMJPH* 2026; 4:e003480. doi: 10.1136/bmjph-2025-003480

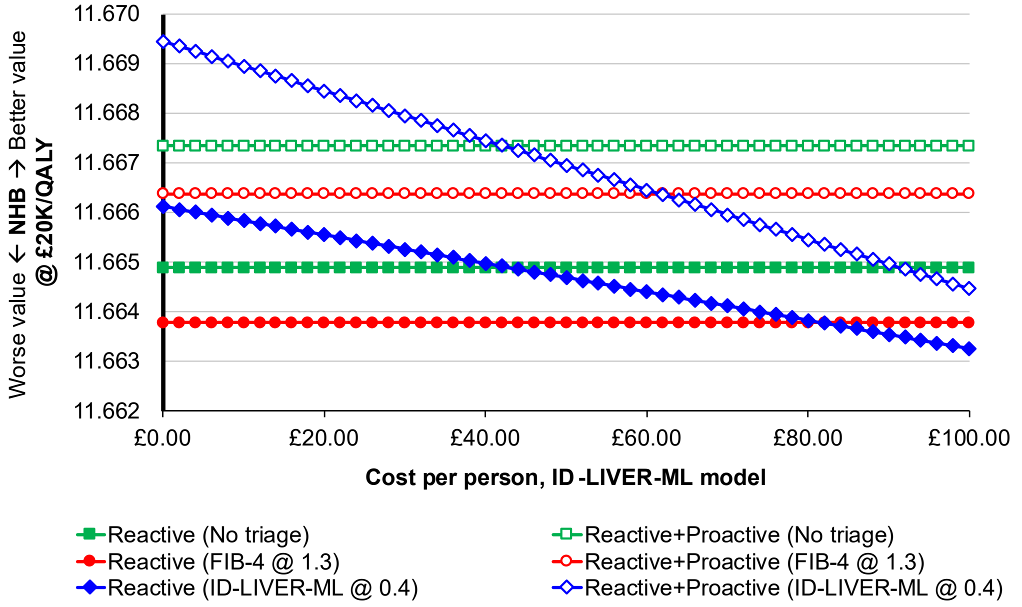

FIB-4 = Fibrosis-4 index; ID-L-ML = ID-LIVER machine-learning risk-prediction model; NHB @ £20K/QALY = net health benefit when we value quality-adjusted life-years at £20,000 each

Figure e6 Threshold analysis: relationship between cost per person of ID-LIVER-ML model and value for money (net health benefit)

Rogers G, *et al. BMJPH* 2026; 4:e003480. doi: 10.1136/bmjph-2025-003480

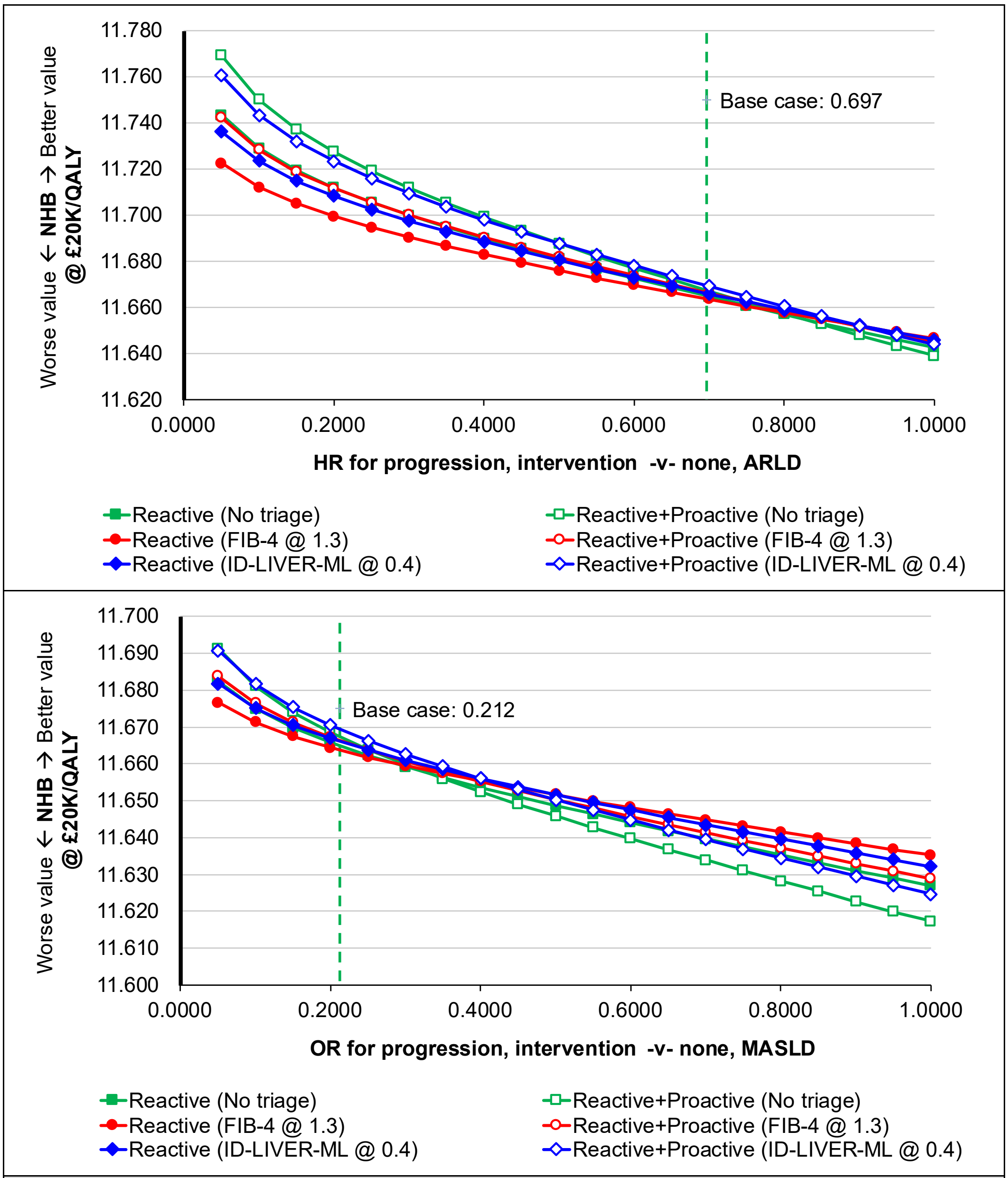

ARLD = alcohol-related liver disease; FIB-4 = Fibrosis-4 index; HR = hazard ratio; ID-L-ML = ID-LIVER machine-learning risk-prediction model; MASLD = metabolic-dysfunction-associated steatotic liver disease; NHB @ £20K/QALY = net health benefit (valuing QALYs at £20,000 each); OR = odds ratio; QALYs = quality-adjusted life-years

Upper panel shows results when varying effectiveness of lifestyle intervention for people with ARLD.
- *Reactive+Proactive (No triage) generates greatest net benefit when HR ≤ 0.45*
- *Reactive+Proactive (ID-LIVER-ML @ 0.4) generates greatest net benefit when 0.5 ≤ HR ≤ 0.85*
- *Reactive (ID-LIVER-ML @ 0.4) generates greatest net benefit when HR = 0.90*
- *Reactive (FIB-4 @ 1.3) generates greatest net benefit when HR ≥ 0.95*

Lower panel shows results when varying effectiveness of lifestyle intervention for people with MASLD.
- *Reactive+Proactive (No triage) generates greatest net benefit when HR = 0.05*
- *Reactive+Proactive (ID-LIVER-ML @ 0.4) generates greatest net benefit when 0.05 < HR ≤ 0.4*
- *Reactive (ID-LIVER-ML @ 0.4) generates greatest net benefit when HR = 0.45*
- *Reactive (FIB-4 @ 1.3) generates greatest net benefit when HR ≥ 0.50*

Figure e7 Threshold analysis: relationship between effectiveness of lifestyle interventions and value for money (net health benefit)

Rogers G, *et al. BMJPH* 2026; 4:e003480. doi: 10.1136/bmjph-2025-003480

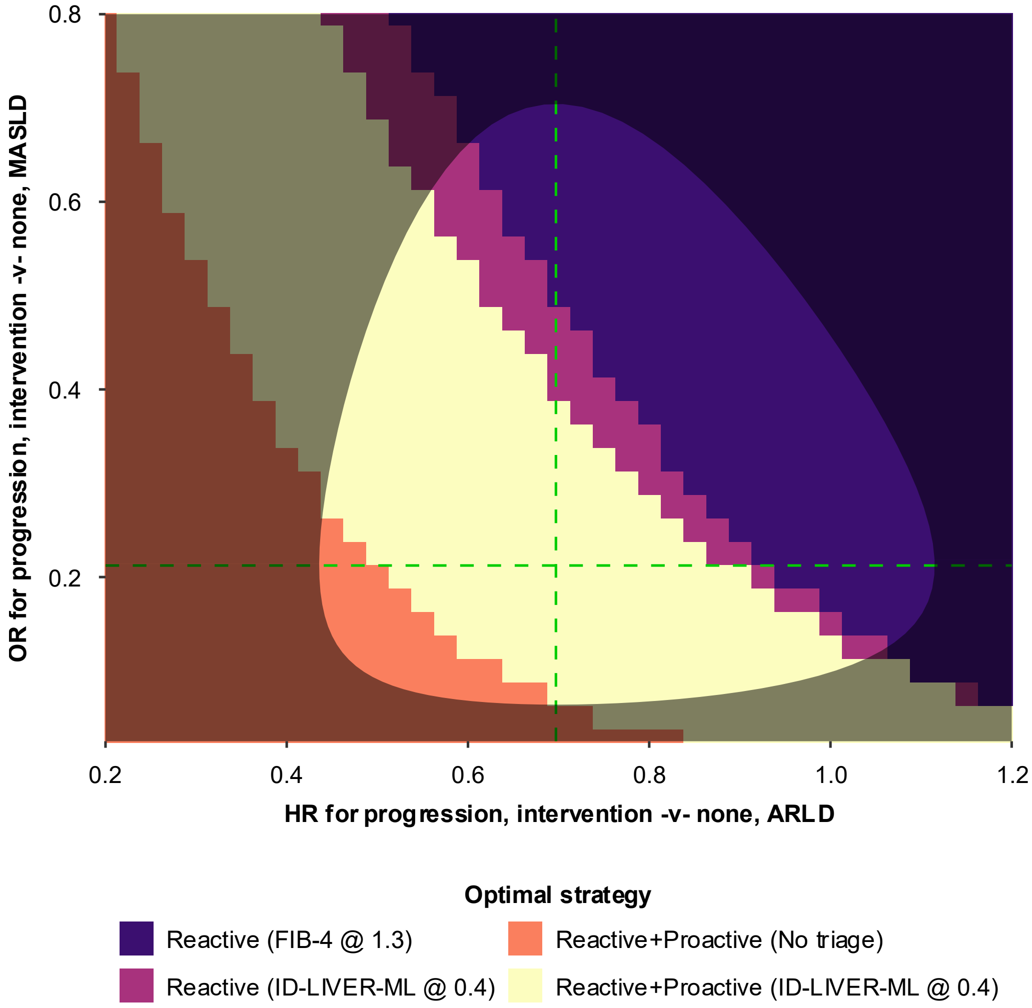

ARLD = alcohol-related liver disease; FIB-4 = Fibrosis-4 index; HR = hazard ratio; ID-LIVER-ML = ID-LIVER machine-learning risk-prediction model; MASLD = metabolic-dysfunction-associated steatotic liver disease; OR = odds ratio

Dashed lines show base-case values for each parameter.

Darker-shaded area shows values outside joint 95% confidence region for the two parameters (assuming no correlation).

Optimal strategy judged in terms of net health benefit (valuing quality-adjusted life-years at £20,000 each);

Figure e8 Two-way sensitivity analysis: relationship between effectiveness of lifestyle interventions and value for money (net health benefit)

eAppendix 10 Probabilistic sensitivity analysis

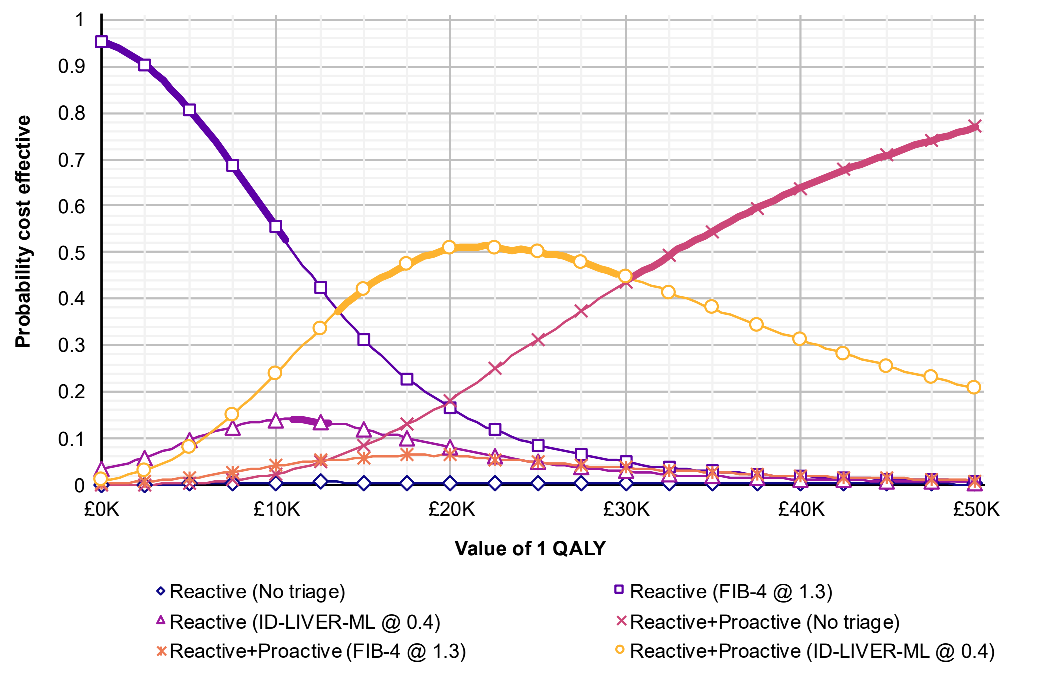

Bold line shows cost-effectiveness acceptability frontier

Figure e9 Cost-effectiveness acceptability curve and frontier

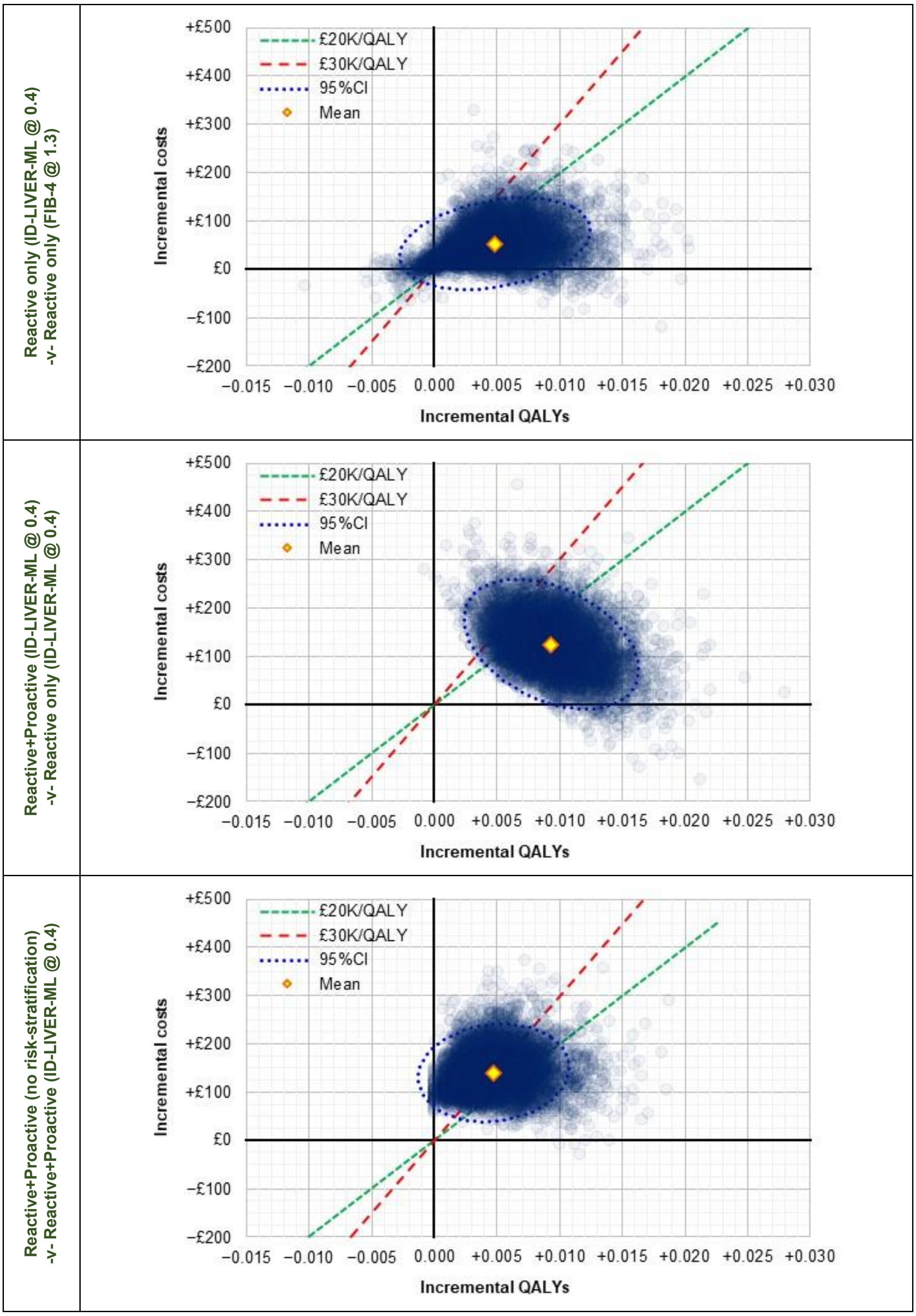

Figure e10 **Scatterplots showing incremental costs and QALYs for key pairwise comparisons in probabilistic analysis (10,000 iterations)**

Rogers G, *et al. BMJPH* 2026; 4:e003480. doi: 10.1136/bmjph-2025-003480

eAppendix 11 One-way sensitivity analysis

We focus, here, on the three sequential pairwise comparisons on the cost-effectiveness frontier in our base case, as these are the comparisons on which our decision uncertainty rests:

1. Compared with the cheapest thing we can do (FIB-4 @ 1.3 in the reactive-only population), is it worth paying extra money to gain more QALYs by using ID-LIVER-ML @ 0.4 instead (*Figure e11*)?
2. Is it good value to extend coverage from the reactive-only population to introduce proactive case-finding in the community (*Figure e12*)?
3. Can we afford to remove risk-stratification so that secondary-care services review all people identified as at risk of CLD, which is guaranteed to maximise QALYs, but will also incur greater costs (*Figure e13*)?

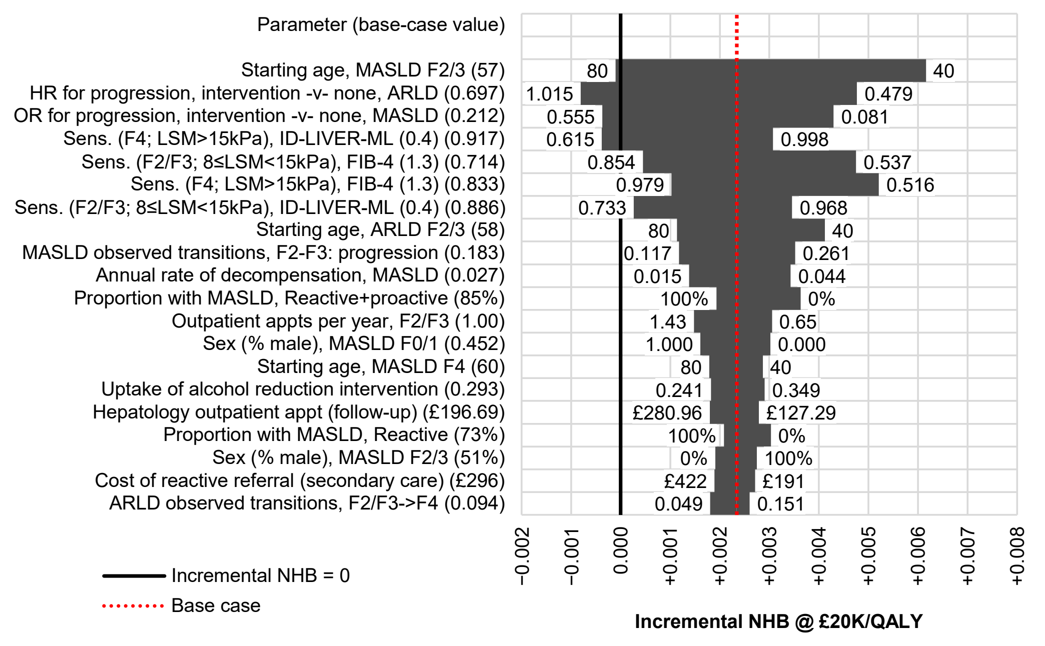

Appt. = appointment; ARLD = alcohol-related liver disease; F0/1/2/3/4 = METAVIR fibrosis stage; HR = hazard ratio; LSM = liver stiffness measurement; MASLD = metabolic-dysfunction-associated steatotic liver disease; NHB = net health benefit; OR = odds ratio; Sens. = sensitivity; Spec. = specificity

Positive incremental NHB values favour ID-LIVER-ML; negative values favour FIB-4. Most influential 20 parameters shown.

Figure e11 One-way sensitivity analysis, reactive-only (ID-LIVER-ML @ 0.4) -v- reactive-only (FIB-4 @ 1.3)

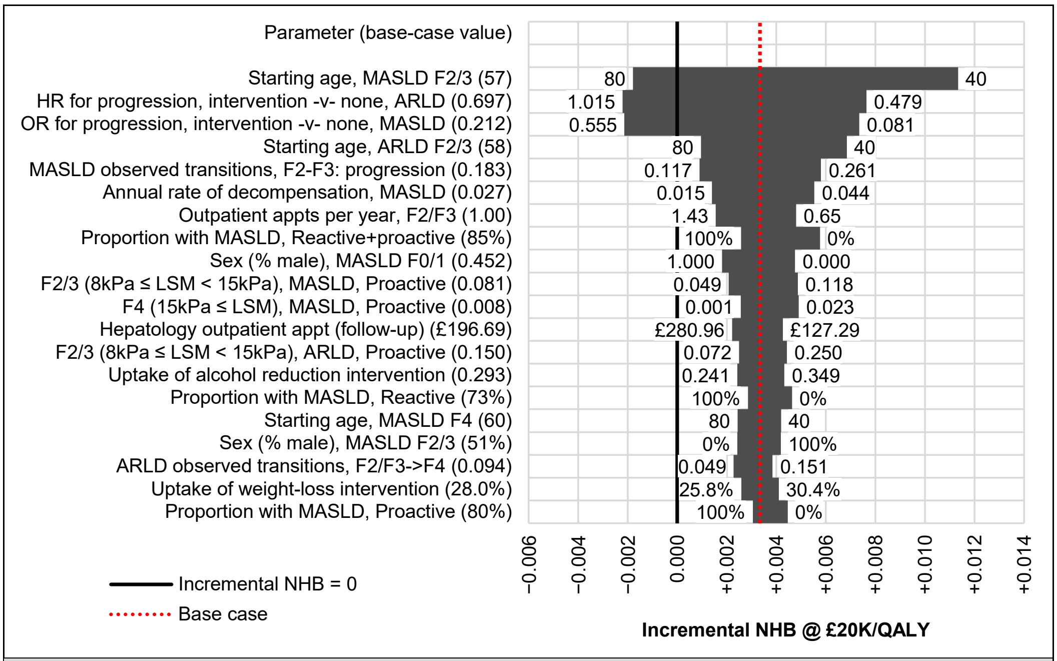

Appt. = appointment; ARLD = alcohol-related liver disease; F0/1/2/3/4 = METAVIR fibrosis stage; HR = hazard ratio; LSM = liver stiffness measurement; MASLD = metabolic-dysfunction-associated steatotic liver disease; NHB = net health benefit; OR = odds ratio; Sens. = sensitivity; Spec. = specificity

Positive incremental NHB values favour ID-LIVER-ML; negative values favour FIB-4. Most influential 20 parameters shown.

Figure e12 One-way sensitivity analysis, reactive+proactive (ID-LIVER-ML @ 0.4) -v- reactive-only (ID-LIVER-ML @ 0.4)

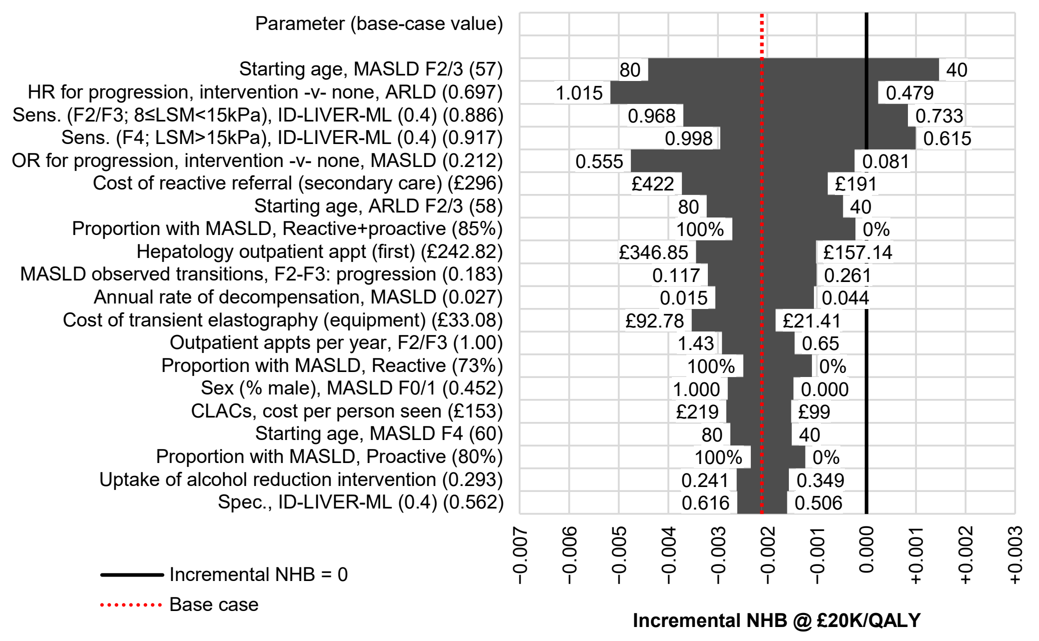

Appt. = appointment; ARLD = alcohol-related liver disease; F0/1/2/3/4 = METAVIR fibrosis stage; HR = hazard ratio; LSM = liver stiffness measurement; MASLD = metabolic-dysfunction-associated steatotic liver disease; NHB = net health benefit; OR = odds ratio; Sens. = sensitivity; Spec. = specificity

Positive incremental NHB values favour ID-LIVER-ML; negative values favour FIB-4. Most influential 20 parameters shown.

Figure e13 One-way sensitivity analysis, reactive+proactive (no risk-stratification) -v- reactive+proactive (ID-LIVER-ML @ 0.4)

eAppendix 12 Scenario analyses

12.1. Removing risk-stratification from proactive case-finding

When we simulate a combination of proactive case-finding and risk-stratification (with FIB-4 or ID-LIVER-ML) in our base case, we effectively assume that data will be available to calculate risk scores for the people identified as at risk of CLD in digital searches of primary care records. This may not always be true, as both risk-stratification tools require reasonably up-to-date inputs (e.g. liver function tests). In the reactive setting, it is reasonable to assume these will always be available, as the referral comes following consultation with primary care clinicians who will invariably order any necessary tests and provide relevant information. However, people in the proactive population, whose risk factors have been identified without any particular interaction with healthcare providers, may not have up-to-date information from which to calculate a risk score. Therefore, we undertook a scenario analysis in which we assumed risk-stratification tools are only relevant to the reactive population, while people in the proactive population will always have to attend a CLAC in order to undergo assessment. Figure illustrates our revised approach to defining the available strategies (cf. *Figure 1* in the main text).

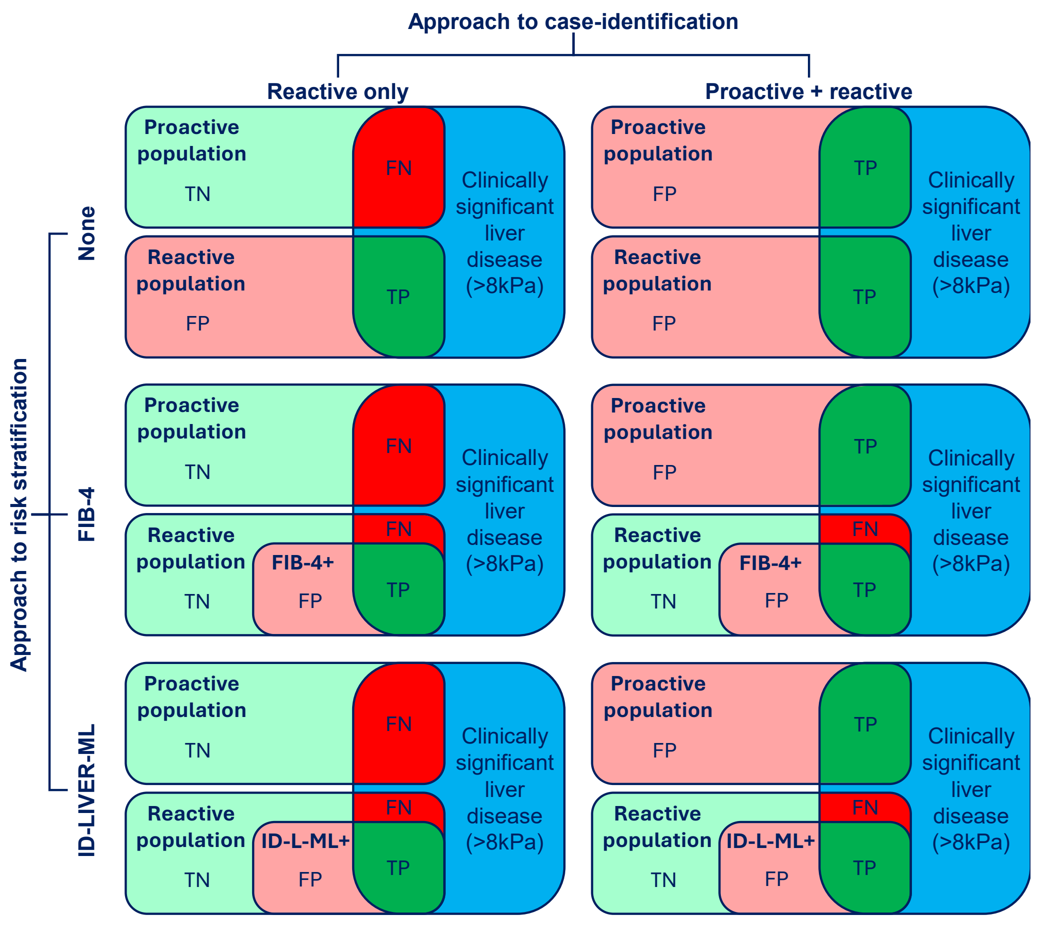

FIB-4 = Fibrosis-4 index; FN = false negative; FP = false positive; ID-L-ML = ID-LIVER machine-learning risk-prediction model; TN = true negative; TP = true positive

Figure e14 Illustration of the six strategies evaluated, combining two approaches to case-identification and three approaches to risk-stratification, when risk-stratification is not possible for the proactive population

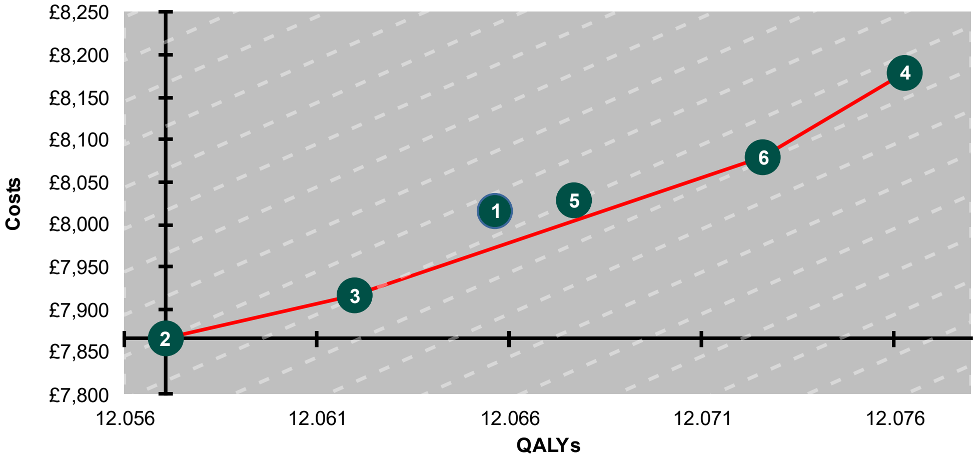

(1) Reactive (No triage)
(2) Reactive (FIB-4 @ 1.3)
(3) Reactive (ID-LIVER-ML @ 0.4)
(4) Reactive+Proactive (No triage)
(5) Reactive+Proactive (FIB-4 @ 1.3)
(6) Reactive+Proactive (ID-LIVER-ML @ 0.4)

Strategy		Strategy-level sensitivity	Absolute		Incremental			Net health benefit	
Case-finding	Risk-stratification (threshold)		Costs (£)	Effects (QALYs)	Costs (£)	Effects (QALYs)	ICER (£/QALY)	£20K/ QALY	£30K/ QALY
Reactive	FIB-4 (1.3)	56.4%	£7,865	12.057				11.664	11.795
Reactive	ID-LIVER-ML (0.4)	67.5%	£7,917	12.062	£52	0.005	£10,498	11.666	11.798
Reactive	None	75.4%	£8,015	12.066	£98	0.004	ext. dom.	11.665	11.798
Reactive+Proactive	FIB-4 (1.3)	81.0%	£8,029	12.068	£111	0.006	ext. dom.	11.666	11.800
Reactive+Proactive	ID-LIVER-ML (0.4)	92.0%	£8,080	12.073	£163	0.011	£15,372	11.669	11.803
Reactive+Proactive	None	100.0%	£8,179	12.076	£98	0.004	£26,729	11.667	11.804

Ext. dom. = extendedly dominated; FIB-4 = Fibrosis-4 index; ID-LIVER-ML = ID-LIVER machine-learning risk-prediction model; QALY = quality-adjusted life-years

Solid red line shows cost-effectiveness frontier. Dashed line in background shows iso-net-benefit gradient equal to £20,000 per QALY. If the line between any two points is shallower than this gradient, the option on the right (generating most QALYs) represents good value; if it is steeper, the option on the left is better value, assuming we value QALYs at £20,000 each.

Sub-table shows fully incremental analysis (i.e. incremental values reflect the comparison between each option and the next-cheapest non-dominated alternative). Strategy-level sensitivity is the proportion of people with significant CLD recruited across reactive and proactive populations in ID-LIVER who would be identified by the strategy in question.

All outcomes represent expected values per person within the whole at-risk population. All outcomes discounted at 3.5% per year.

Figure e15 Scenario analysis removing risk-stratification from proactive case-finding: cost-effectiveness results

12.2. Limiting effectiveness of lifestyle interventions to single transitions that most closely reflect context in which the underlying studies collected data

In this scenario analysis, we only apply the benefits of lifestyle interventions to the transitions that most closely reflect the data on which they are based (for ARLD, only affecting the

transition from compensated → decompensated cirrhosis; for MASLD, only affecting movement between fibrosis states).

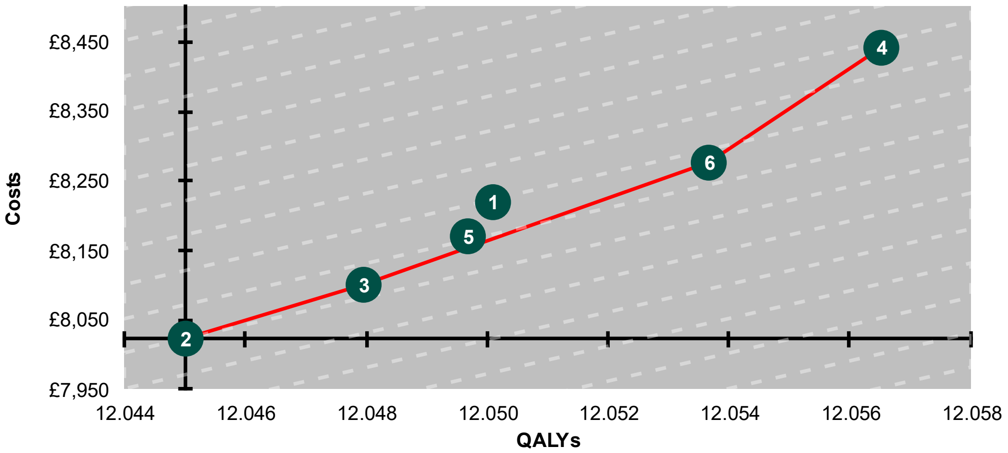

(1) Reactive (No triage)
(2) Reactive (FIB-4 @ 1.3)
(3) Reactive (ID-LIVER-ML @ 0.4)
(4) Reactive+Proactive (No triage)
(5) Reactive+Proactive (FIB-4 @ 1.3)
(6) Reactive+Proactive (ID-LIVER-ML @ 0.4)

Strategy		Strategy-level sensitivity	Absolute		Incremental			Net health benefit	
Case-finding	Risk-stratification (threshold)		Costs (£)	Effects (QALYs)	Costs (£)	Effects (QALYs)	ICER (£/QALY)	£20K/ QALY	£30K/ QALY
Reactive	FIB-4 (1.3)	56.4%	£8,022	12.045				11.644	11.778
Reactive	ID-LIVER-ML (0.4)	67.5%	£8,101	12.048	£79	0.003	£26,893	11.643	11.778
Reactive+Proactive	FIB-4 (1.3)	74.3%	£8,170	12.050	£69	0.002	ext. dom.	11.641	11.777
Reactive	None	75.4%	£8,220	12.050	£119	0.002	ext. dom.	11.639	11.776
Reactive+Proactive	ID-LIVER-ML (0.4)	89.3%	£8,277	12.054	£175	0.006	£30,713	11.640	11.778
Reactive+Proactive	None	100.0%	£8,443	12.057	£166	0.003	£58,270	11.634	11.775

Ext. dom. = extendedly dominated; FIB-4 = Fibrosis-4 index; ID-LIVER-ML = ID-LIVER machine-learning risk-prediction model; QALY = quality-adjusted life-years

Solid red line shows cost-effectiveness frontier. Dashed line in background shows iso-net-benefit gradient equal to £20,000 per QALY. If the line between any two points is shallower than this gradient, the option on the right (generating most QALYs) represents good value; if it is steeper, the option on the left is better value, assuming we value QALYs at £20,000 each.

Sub-table shows fully incremental analysis (i.e. incremental values reflect the comparison between each option and the next-cheapest non-dominated alternative). Strategy-level sensitivity is the proportion of people with significant CLD recruited across reactive and proactive populations in ID-LIVER who would be identified by the strategy in question.

All outcomes represent expected values per person within the whole at-risk population. All outcomes discounted at 3.5% per year.

Figure e16 Scenario analysis limiting effectiveness of lifestyle interventions to single transitions that most closely reflect context in which the underlying studies collected data: cost-effectiveness results

12.3. Assuming health-related quality of life changes with F-stage in people with predominantly asymptomatic disease

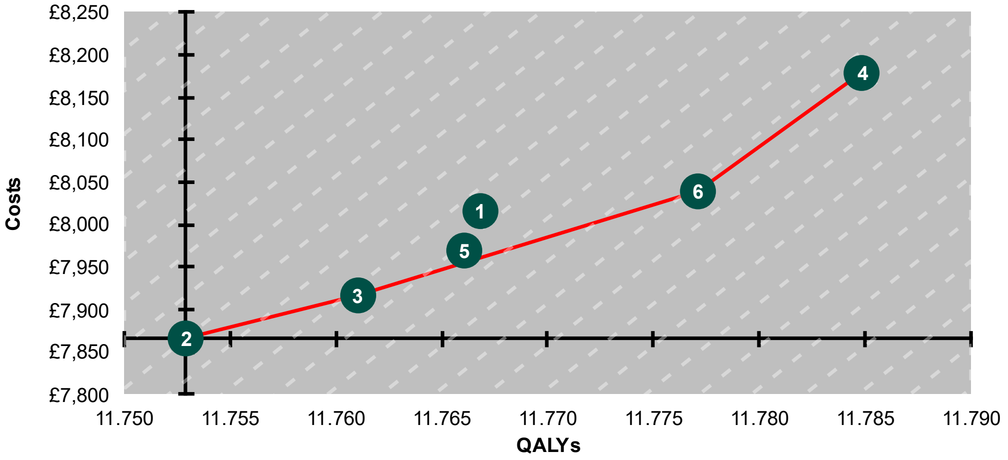

(1) Reactive (No triage)
(2) Reactive (FIB-4 @ 1.3)
(3) Reactive (ID-LIVER-ML @ 0.4)
(4) Reactive+Proactive (No triage)
(5) Reactive+Proactive (FIB-4 @ 1.3)
(6) Reactive+Proactive (ID-LIVER-ML @ 0.4)

Strategy		Strategy-level sensitivity	Absolute		Incremental			Net health benefit	
Case-finding	Risk-stratification (threshold)		Costs (£)	Effects (QALYs)	Costs (£)	Effects (QALYs)	ICER (£/QALY)	£20K/ QALY	£30K/ QALY
Reactive	FIB-4 (1.3)	56.4%	£7,865	11.753				11.360	11.491
Reactive	ID-LIVER-ML (0.4)	67.5%	£7,917	11.761	£52	0.008	£6,389	11.365	11.497
Reactive+Proactive	FIB-4 (1.3)	74.3%	£7,970	11.766	£52	0.005	ext. dom.	11.368	11.500
Reactive	None	75.4%	£8,015	11.767	£98	0.006	ext. dom.	11.366	11.500
Reactive+Proactive	ID-LIVER-ML (0.4)	89.3%	£8,040	11.777	£123	0.016	£7,624	11.375	11.509
Reactive+Proactive	None	100.0%	£8,179	11.785	£139	0.008	£17,810	11.376	11.512

Ext. dom. = extendedly dominated; FIB-4 = Fibrosis-4 index; ID-LIVER-ML = ID-LIVER machine-learning risk-prediction model; QALY = quality-adjusted life-years

Solid red line shows cost-effectiveness frontier. Dashed line in background shows iso-net-benefit gradient equal to £20,000 per QALY. If the line between any two points is shallower than this gradient, the option on the right (generating most QALYs) represents good value; if it is steeper, the option on the left is better value, assuming we value QALYs at £20,000 each.

Sub-table shows fully incremental analysis (i.e. incremental values reflect the comparison between each option and the next-cheapest non-dominated alternative). Strategy-level sensitivity is the proportion of people with significant CLD recruited across reactive and proactive populations in ID-LIVER who would be identified by the strategy in question.

All outcomes represent expected values per person within the whole at-risk population. All outcomes discounted at 3.5% per year.

Figure e17 Scenario analysis assuming health-related quality of life changes with F-stage in people with predominantly asymptomatic disease: cost-effectiveness results

Rogers G, *et al. BMJPH* 2026; 4:e003480. doi: 10.1136/bmjph-2025-003480

12.4. Expanding the intervention for ARLD to include pharmacotherapy in addition to behavioural intervention

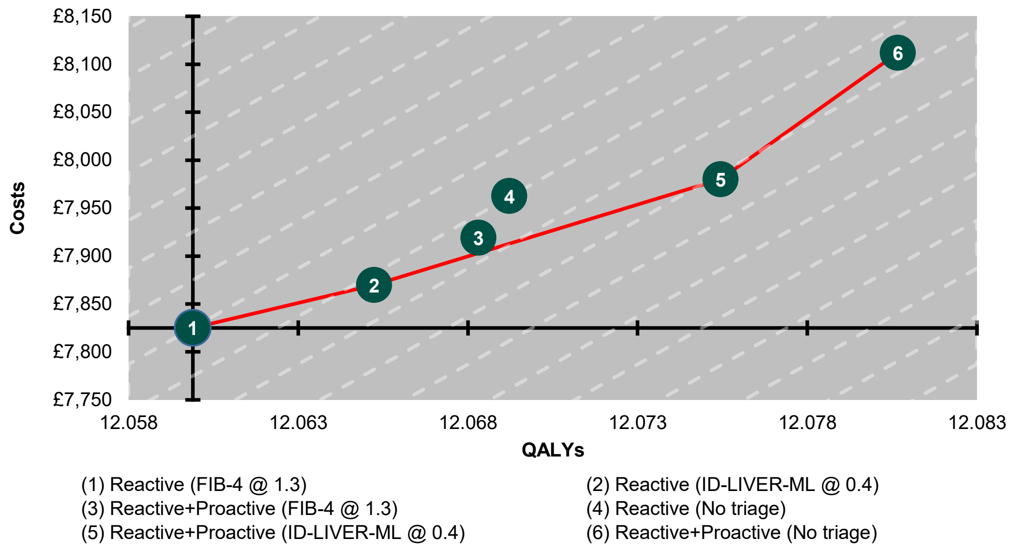

Strategy		Strategy-level sensitivity	Absolute		Incremental			Net health benefit	
Case-finding	Risk-stratification (threshold)		Costs (£)	Effects (QALYs)	Costs (£)	Effects (QALYs)	ICER (£/QALY)	£20K/ QALY	£30K/ QALY
Reactive	FIB-4 (1.3)	56.4%	£7,825	12.059				11.668	11.799
Reactive	ID-LIVER-ML (0.4)	67.5%	£7,870	12.064	£45	0.005	£8,366	11.671	11.802
Reactive+Proactive	FIB-4 (1.3)	74.3%	£7,919	12.068	£49	0.003	ext. dom.	11.672	11.804
Reactive	None	75.4%	£7,962	12.069	£93	0.004	ext. dom.	11.671	11.803
Reactive+Proactive	ID-LIVER-ML (0.4)	89.3%	£7,980	12.075	£110	0.010	£10,805	11.676	11.809
Reactive+Proactive	None	100.0%	£8,112	12.080	£132	0.005	£25,234	11.675	11.810

Ext. dom. = extendedly dominated; FIB-4 = Fibrosis-4 index; ID-LIVER-ML = ID-LIVER machine-learning risk-prediction model; QALY = quality-adjusted life-years

Solid red line shows cost-effectiveness frontier. Dashed line in background shows iso-net-benefit gradient equal to £20,000 per QALY. If the line between any two points is shallower than this gradient, the option on the right (generating most QALYs) represents good value; if it is steeper, the option on the left is better value, assuming we value QALYs at £20,000 each.

Sub-table shows fully incremental analysis (i.e. incremental values reflect the comparison between each option and the next-cheapest non-dominated alternative). Strategy-level sensitivity is the proportion of people with significant CLD recruited across reactive and proactive populations in ID-LIVER who would be identified by the strategy in question.

All outcomes represent expected values per person within the whole at-risk population. All outcomes discounted at 3.5% per year.

Figure e18 Scenario analysis assuming the intervention for ARLD is expanded to incorporate medication alongside behavioural intervention

12.5. Estimating the long-term effectiveness of lifestyle modification for MASLD using a 9-month effect

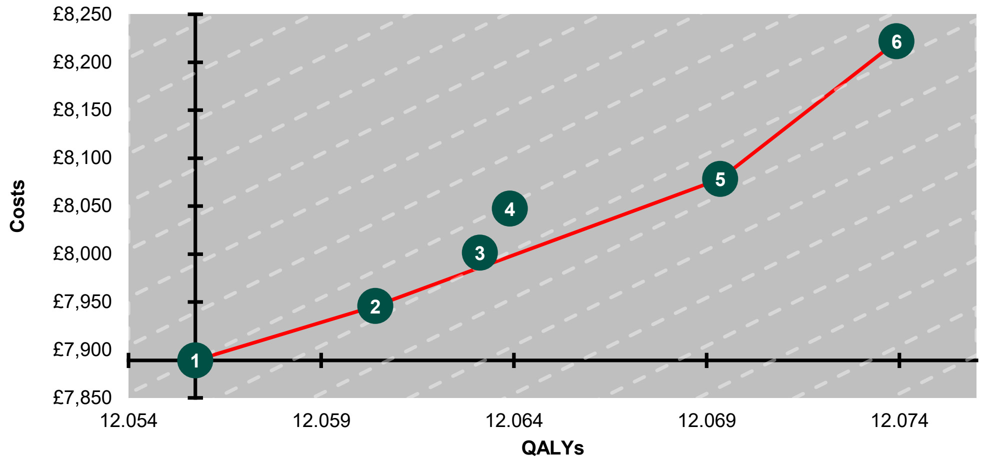

(1) Reactive (FIB-4 @ 1.3)
(2) Reactive (ID-LIVER-ML @ 0.4)
(3) Reactive+Proactive (FIB-4 @ 1.3)
(4) Reactive (No triage)
(5) Reactive+Proactive (ID-LIVER-ML @ 0.4)
(6) Reactive+Proactive (No triage)

Strategy		Strategy-level sensitivity	Absolute		Incremental			Net health benefit	
Case-finding	Risk-stratification (threshold)		Costs (£)	Effects (QALYs)	Costs (£)	Effects (QALYs)	ICER (£/QALY)	£20K/ QALY	£30K/ QALY
Reactive	FIB-4 (1.3)	56.4%	£7,889	12.056				11.661	11.793
Reactive	ID-LIVER-ML (0.4)	67.5%	£7,946	12.060	£57	0.005	£12,124	11.663	11.796
Reactive+Proactive	FIB-4 (1.3)	74.3%	£8,001	12.063	£56	0.003	ext. dom.	11.663	11.796
Reactive	None	75.4%	£8,047	12.064	£102	0.004	ext. dom.	11.662	11.796
Reactive+Proactive	ID-LIVER-ML (0.4)	89.3%	£8,078	12.069	£133	0.009	£14,821	11.665	11.800
Reactive+Proactive	None	100.0%	£8,222	12.074	£143	0.005	£31,349	11.663	11.800

Ext. dom. = extendedly dominated; FIB-4 = Fibrosis-4 index; ID-LIVER-ML = ID-LIVER machine-learning risk-prediction model; QALY = quality-adjusted life-years

Solid red line shows cost-effectiveness frontier. Dashed line in background shows iso-net-benefit gradient equal to £20,000 per QALY. If the line between any two points is shallower than this gradient, the option on the right (generating most QALYs) represents good value; if it is steeper, the option on the left is better value, assuming we value QALYs at £20,000 each.

Sub-table shows fully incremental analysis (i.e. incremental values reflect the comparison between each option and the next-cheapest non-dominated alternative). Strategy-level sensitivity is the proportion of people with significant CLD recruited across reactive and proactive populations in ID-LIVER who would be identified by the strategy in question.

All outcomes represent expected values per person within the whole at-risk population. All outcomes discounted at 3.5% per year.

Figure e19 Scenario analysis estimating long-term effectiveness of lifestyle modification for MASLD using 9-month effect

Rogers G, et al. BMJPH 2026; 4:e003480. doi: 10.1136/bmjph-2025-003480

12.6. Costing proactive case-finding using costs assigned during ID-LIVER research project

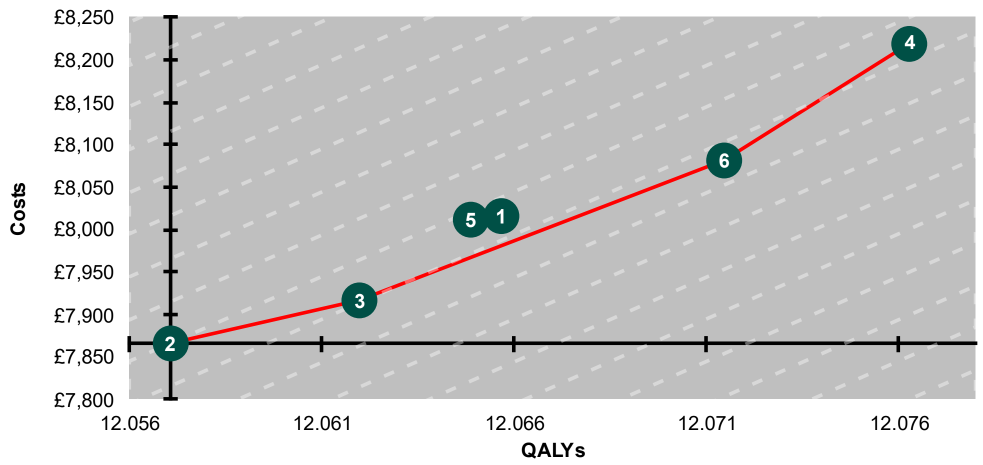

(1) Reactive (No triage)
(2) Reactive (FIB-4 @ 1.3)
(3) Reactive (ID-LIVER-ML @ 0.4)
(4) Reactive+Proactive (No triage)
(5) Reactive+Proactive (FIB-4 @ 1.3)
(6) Reactive+Proactive (ID-LIVER-ML @ 0.4)

Strategy		Strategy-level sensitivity	Absolute		Incremental			Net health benefit	
Case-finding	Risk-stratification (threshold)		Costs (£)	Effects (QALYs)	Costs (£)	Effects (QALYs)	ICER (£/QALY)	£20K/ QALY	£30K/ QALY
Reactive	FIB-4 (1.3)	56.4%	£7,865	12.057				11.664	11.795
Reactive	ID-LIVER-ML (0.4)	67.5%	£7,917	12.062	£52	0.005	£10,498	11.666	11.798
Reactive+Proactive	FIB-4 (1.3)	74.3%	£8,011	12.065	£94	0.003	ext. dom.	11.664	11.798
Reactive	None	75.4%	£8,015	12.066	£98	0.004	ext. dom.	11.665	11.798
Reactive+Proactive	ID-LIVER-ML (0.4)	89.3%	£8,081	12.071	£164	0.009	£17,340	11.667	11.802
Reactive+Proactive	None	100.0%	£8,220	12.076	£139	0.005	£28,753	11.665	11.802

Ext. dom. = extendedly dominated; FIB-4 = Fibrosis-4 index; ID-LIVER-ML = ID-LIVER machine-learning risk-prediction model; QALY = quality-adjusted life-years

Solid red line shows cost-effectiveness frontier. Dashed line in background shows iso-net-benefit gradient equal to £20,000 per QALY. If the line between any two points is shallower than this gradient, the option on the right (generating most QALYs) represents good value; if it is steeper, the option on the left is better value, assuming we value QALYs at £20,000 each.

Sub-table shows fully incremental analysis (i.e. incremental values reflect the comparison between each option and the next-cheapest non-dominated alternative). Strategy-level sensitivity is the proportion of people with significant CLD recruited across reactive and proactive populations in ID-LIVER who would be identified by the strategy in question.

All outcomes represent expected values per person within the whole at-risk population. All outcomes discounted at 3.5% per year.

Figure e20 Scenario analysis using costs assigned to proactive case-finding during ID-LIVER research project

Rogers G, *et al. BMJPH* 2026; 4:e003480. doi: 10.1136/bmjph-2025-003480

References for eAppendices

1. Nakano M, Worner TM, Lieber CS. Perivenular Fibrosis in Alcoholic Liver Injury: Ultrastructure and Histologic Progression. Gastroenterology. 1982;83(4):777–85. doi:10.1016/S0016-5085(82)80006-1 PubMed PMID: 7106508.

2. Marbet UA, Bianchi L, Meury U, Stalder GA. Long-term histological evaluation of the natural history and prognostic factors of alcoholic liver disease. J Hepatol. 1987;4(3):364–72. doi:10.1016/S0168-8278(87)80547-0

3. Worner TM, Lieber CS. Perivenular Fibrosis as Precursor Lesion of Cirrhosis. JAMA. 1985;254(5):627–30. doi:10.1001/jama.1985.03360050065027

4. Mcpherson S, Pais R, Valenti L, Schattenberg JM, Dufour JF, Tsochatzis E, et al. Further delineation of fibrosis progression in NAFLD: evidence from a large cohort of patients with sequential biopsies. J Hepatol. 2017;66(1):S593. doi:10.1016/S0168-8278(17)31613-6

5. Fleming KM, Aithal GP, Card TR, West J. The rate of decompensation and clinical progression of disease in people with cirrhosis: a cohort study. Aliment Pharmacol Ther. 2010;32(11–12):1343–50. doi:10.1111/j.1365-2036.2010.04473.x

6. Sanyal AJ, Natta MLV, Clark J, Neuschwander-Tetri BA, Diehl A, Dasarathy S, et al. Prospective Study of Outcomes in Adults with Nonalcoholic Fatty Liver Disease. N Engl J Med. 2021;385(17):1559–69. doi:10.1056/NEJMoa2029349

7. West J, Card TR, Aithal GP, Fleming KM. Risk of hepatocellular carcinoma among individuals with different aetiologies of cirrhosis: a population-based cohort study. Aliment Pharmacol Ther. 2017;45(7):983–90. doi:10.1111/apt.13961

8. Huang DQ, Tran S, Barnett S, Zou B, Yeo YH, Cheung R, et al. Incidence and predictors of hepatocellular carcinoma in NAFLD without diagnosed cirrhosis: a nationwide real-world U.S. study. Hepatol Int. 2024;18(2):540–9. doi:10.1007/s12072-023-10616-8

9. Reddy KR, McLerran D, Marsh T, Parikh N, Roberts LR, Schwartz M, et al. Incidence and Risk Factors for Hepatocellular Carcinoma in Cirrhosis: The Multicenter Hepatocellular Carcinoma Early Detection Strategy (HEDS) Study. Gastroenterology. 2023;165(4):1053-1063.e6. doi:10.1053/j.gastro.2023.06.027 PubMed PMID: 37429366.

10. Bengtsson B, Widman L, Wahlin S, Stål P, Björkström NK, Hagström H. The risk of hepatocellular carcinoma in cirrhosis differs by etiology, age and sex: A Swedish nationwide population-based cohort study. United Eur Gastroenterol J. 2022;10(5):465–76. doi:10.1002/ueg2.12238

11. Orci LA, Sanduzzi-Zamparelli M, Caballol B, Sapena V, Colucci N, Torres F, et al. Incidence of Hepatocellular Carcinoma in Patients With Nonalcoholic Fatty Liver Disease: A Systematic Review, Meta-analysis, and Meta-regression. Clin Gastroenterol Hepatol. 2022;20(2):283-292.e10. doi:10.1016/j.cgh.2021.05.002 PubMed PMID: 33965578.

12. Fleming KM, Aithal GP, Card TR, West J. All-cause mortality in people with cirrhosis compared with the general population: a population-based cohort study. Liver Int. 2012;32(1):79–84. doi:10.1111/j.1478-3231.2011.02517.x

13. Davila JA, Morgan RO, Richardson PA, Du XL, McGlynn KA, El-Serag HB. Use of surveillance for hepatocellular carcinoma among patients with cirrhosis in the United States. Hepatology. 2010;52(1):132–41. doi:10.1002/hep.23615

14. Scragg J, Avery L, Cassidy S, Taylor G, Haigh L, Boyle M, et al. Feasibility of a Very Low Calorie Diet to Achieve a Sustainable 10% Weight Loss in Patients With Nonalcoholic Fatty Liver Disease. Clin Transl Gastroenterol. 2020;11(9):e00231. doi:10.14309/ctg.0000000000000231

15. Taylor R, Leslie WS, Barnes AC, Brosnahan N, Thom G, McCombie L, et al. Clinical and metabolic features of the randomised controlled Diabetes Remission Clinical Trial (DiRECT) cohort. Diabetologia. 2018;61(3):589–98. doi:10.1007/s00125-017-4503-0

16. Rogal S, Youk A, Zhang H, Gellad WF, Fine MJ, Good CB, et al. Impact of Alcohol Use Disorder Treatment on Clinical Outcomes Among Patients With Cirrhosis. Hepatology. 2020;71(6):2080. doi:10.1002/hep.31042

17. Vannier AGL, Przybyszewski EM, Shay J, Patel SJ, Schaefer E, Goodman RP, et al. Psychotherapy for Alcohol Use Disorder Is Associated With Reduced Risk of Incident Alcohol-Associated Liver Disease. Clin Gastroenterol Hepatol. 2023;21(6):1571-1580.e7. doi:10.1016/j.cgh.2022.08.001

18. Mellinger JL, Fernandez A, Shedden K, Winder GS, Fontana RJ, Volk ML, et al. Gender Disparities in Alcohol Use Disorder Treatment Among Privately Insured Patients with Alcohol-Associated Cirrhosis. Alcohol Clin Exp Res. 2019;43(2):334–41. doi:10.1111/acer.13944

19. Crawford MJ, Patton R, Touquet R, Drummond C, Byford S, Barrett B, et al. Screening and referral for brief intervention of alcohol-misusing patients in an emergency department: a pragmatic randomised controlled trial. The Lancet. 2004;364(9442):1334–9. doi:10.1016/S0140-6736(04)17190-0 PubMed PMID: 15474136.

20. Haq MI, Drake TM, Goh TL, Ahmed A, Forrest E, Barclay S, et al. Effect of Hepatocellular Carcinoma Surveillance Programmes on Overall Survival in a Mixed Cirrhotic UK Population: A Prospective, Longitudinal Cohort Study. J Clin Med. 2021;10(13):13. doi:10.3390/jcm10132770

21. Cullen K, Jones M, Pockett RD, Burton A, Cross TJS, Rowe IA, et al. Cost of hepatocellular carcinoma to the national health service in England: a registry-based analysis. BMJ Open Gastroenterol. 2023;10(1):e000998. doi:10.1136/bmjgast-2022-000998 PubMed PMID: 36810207.

22. NHS England. 2023/24 National Cost Collection Data for the NHS [Internet]. 2024 [cited 2024 Dec 20]. Available from: https://www.england.nhs.uk/publication/2023-24-national-cost-collection-data-publication/

23. Jones KC, Weatherly H, Birch S, Castelli A, Chalkley M, Dargan A, et al. Unit Costs of Health and Social Care 2024 Manual [Reports and Papers] [Internet]. Kent, UK: Personal Social Services Research Unit (University of Kent) & Centre for Health Economics (University of York); 2025 Apr [cited 2025 Apr 12]. Available from: https://www.pssru.ac.uk/unitcostsreport/

24. Hernández Alava M, Pudney S, Wailoo A. Estimating EQ-5D by age and sex for the UK [Internet]. NICE Decision Suport Unit; 2022 Jul [cited 2024 Dec 20]. Available from: https://www.sheffield.ac.uk/nice-dsu/methods-development/estimating-eq-5d

25. Verma M, Paik JM, Younossi I, Tan D, Abdelaal H, Younossi ZM. The impact of hepatocellular carcinoma diagnosis on patients' health-related quality of life. Cancer Med. 2021;10(18):6273–81. doi:10.1002/cam4.4166

26. Parker R, Aithal GP, Becker U, Gleeson D, Masson S, Wyatt JI, et al. Natural history of histologically proven alcohol-related liver disease: A systematic review. J Hepatol. 2019;71(3):586–93. doi:10.1016/j.jhep.2019.05.020

27. Hasselblad V, Hedges LV. Meta-analysis of screening and diagnostic tests. Psychol Bull. 1995;117(1):167–78. doi:10.1037/0033-2909.117.1.167

28. Chinn S. A simple method for converting an odds ratio to effect size for use in meta-analysis. Stat Med. 2000;19(22):3127–31. doi:10.1002/1097-0258(20001130)19:22%3C3127::AID-SIM784%3E3.0.CO;2-M

29. Guyot P, Ades A, Ouwens MJ, Welton NJ. Enhanced secondary analysis of survival data: reconstructing the data from published Kaplan-Meier survival curves. BMC Med Res Methodol. 2012;12(1):9. doi:10.1186/1471-2288-12-9

30. Every Health [Internet]. [cited 2024 Dec 11]. Every Health. Available from: https://www.everyhealth.com/

31. Crawford MJ, Patton R, Touquet R, Drummond C, Byford S, Barrett B, et al. Screening and referral for brief intervention of alcohol-misusing patients in an emergency department: a pragmatic randomised controlled trial. The Lancet. 2004;364(9442):1334–9. doi:10.1016/S0140-6736(04)17190-0

32. Driver RJ. Understanding Outcomes in Hepatocellular Carcinoma and Cirrhosis in England [md] [Internet]. University of Leeds; 2022 [cited 2025 Mar 14]. Available from: https://etheses.whiterose.ac.uk/id/eprint/31228/

33. Hernandez M, Pudney S, Wailoo A. Estimating the relationship between EQ-5D-5L and EQ-5D-3L: results from an English population study [report] [Internet]. The University of Sheffield; 2020 Sep [cited 2024 Dec 20]. Available from: https://orda.shef.ac.uk/articles/report/Estimating_the_relationship_between_EQ-5D-5L_and_EQ-5D-3L_results_from_an_English_population_study/25219157/1 doi:10.15131/shef.data.25219157.v1

34. Björnsson E, Verbaan H, Oksanen A, Frydén A, Johansson J, Friberg S, et al. Health-related quality of life in patients with different stages of liver disease induced by hepatitis C. Scand J Gastroenterol. 2009;44(7):878–87. doi:10.1080/00365520902898135 PubMed PMID: 19437190.

35. Chong CAKY, Gulamhussein A, Heathcote EJ, Lilly L, Sherman M, Naglie G, et al. Health-State Utilities and Quality of Life in Hepatitis C Patients. Am J Gastroenterol. 2003;98(3):630–8. doi:10.1111/j.1572-0241.2003.07332.x

36. Cortesi PA, Conti S, Scalone L, Jaffe A, Ciaccio A, Okolicsanyi S, et al. Health related quality of life in chronic liver diseases. Liver Int. 2020;40(11):2630–42. doi:10.1111/liv.14647

37. Pol S, Chevalier J, Branchoux S, Perry R, Milligan G, Gaudin AF. P0747 : Health related quality of life and utility values in chronic hepatitis C patients: A cross-sectional study in France, the Uk and Germany. J Hepatol. 2015;62:S606. doi:10.1016/S0168-8278(15)30950-8

38. Samp JC, Perry R, Piercy J, Wood R, Baran RW. Patient health utility, work productivity, and lifestyle impairment in chronic hepatitis C patients in France. Clin Res Hepatol Gastroenterol. 2015;39(3):307–14. doi:10.1016/j.clinre.2014.10.013

39. Sugimori H, Hirao M, Igarashi A, Yatsuhashi H, Ikeda S, Masaki N, et al. Health state utilities of patients with hepatitis B and C and hepatitis-related conditions in Japan. Sci Rep. 2022;12:17139. doi:10.1038/s41598-022-21470-3 PubMed PMID: 36229479; PubMed Central PMCID: PMC9561176.

40. Vargas C, Espinoza MA, Giglio A, Soza A. Cost effectiveness of Daclatasvir/Asunaprevir versus Peginterferon/Ribavirin and protease inhibitors for the treatment of Hepatitis C Genotype 1B in Chile. Value Health. 2015;18(3):A225–6. doi:10.1016/j.jval.2015.03.1309

41. Vellopoulou A, van Agthoven M, van der Kolk A, de Knegt RJ, Berdeaux G, Cure S, et al. Cost Utility of Telaprevir–PR (Peginterferon–Ribavirin) Versus Boceprevir–PR and Versus PR Alone in Chronic Hepatitis C in The Netherlands. Appl Health Econ Health Policy. 2014;12(6):647–59. doi:10.1007/s40258-014-0120-y

42. Woo G, Tomlinson G, Yim C, Lilly L, Therapondos G, Wong DK, et al. Health State Utilities and Quality of Life in Patients with Hepatitis B. Can J Gastroenterol Hepatol. NaN/NaN/NaN;26:445–51. doi:10.1155/2012/736452

43. Wright M, Grieve R, Roberts J, Main J, Thomas H. Health benefits of antiviral therapy for mild chronic hepatitis C: randomised controlled trial and economic evaluation. Health Technol Assess. 2006;10(21). doi:10.3310/hta10210

44. Ara R, Brazier J. Deriving an Algorithm to Convert the Eight Mean SF-36 Dimension Scores into a Mean EQ-5D Preference-Based Score from Published Studies (Where Patient Level Data Are Not Available). Value Health. 2008;11(7):1131–43. doi:10.1111/j.1524-4733.2008.00352.x PubMed PMID: 18489495.
